# ANNOgesic: a Swiss army knife for the RNA-seq based annotation of bacterial/archaeal genomes

**DOI:** 10.1093/gigascience/giy096

**Published:** 2018-08-30

**Authors:** Sung-Huan Yu, Jörg Vogel, Konrad U Förstner

**Affiliations:** 1 Institute of Molecular Infection Biology (IMIB), University of Würzburg, Josef-Schneider-Straße 2, 97080 Würzburg, Germany; 2 Helmholtz Institute for RNA-based Infection Research (HIRI), Josef-Schneider-Straße 2, 97080 Würzburg Germany; 3 ZB MED - Information Center for Life Sciences, Informationservices, Gleueler Straße 60, 50931 Cologne (Köln), Germany; 4 Technical University of Cologne, Faculty for Information and Communication Sciences, Claudiusstraße 1, 50678 Cologne (Köln), Germany

**Keywords:** genome annotation, RNA-seq, transcriptomics

## Abstract

To understand the gene regulation of an organism of interest, a comprehensive genome annotation is essential. While some features, such as coding sequences, can be computationally predicted with high accuracy based purely on the genomic sequence, others, such as promoter elements or noncoding RNAs, are harder to detect. RNA sequencing (RNA-seq) has proven to be an efficient method to identify these genomic features and to improve genome annotations. However, processing and integrating RNA-seq data in order to generate high-resolution annotations is challenging, time consuming, and requires numerous steps. We have constructed a powerful and modular tool called ANNOgesic that provides the required analyses and simplifies RNA-seq-based bacterial and archaeal genome annotation. It can integrate data from conventional RNA-seq and differential RNA-seq and predicts and annotates numerous features, including small noncoding RNAs, with high precision. The software is available under an open source license (ISCL) at https://pypi.org/project/ANNOgesic/.

## Background

As the number of available genome sequences has rapidly expanded in databases, numerous tools have been developed that can detect genomic features of interest based on the genome sequence. Prominent representatives are Glimmer to identify open reading frames (ORFs) [1], tRNAscan-SE [2] to spot tRNAs, and RNAmmer to find rRNAs [3]. Pipelines such as Prokka [4] or ConsPred [5] combine such tools and are able to search multiple features in bacterial and archaeal genomes. Still, these tools make their predictions purely based on the genome sequences and can predict features such as transcriptional start sites and noncoding RNAs, if at all, only with low confidence.

Recent developments in high-throughput sequencing offer solutions to this problem. RNA sequencing (RNA-seq) has revolutionized how differential gene expression can be measured and is widely used for this purpose [6]. In addition, it has also been applied in numerous cases to improve the genome annotation of bacteria [7,8,9]archaea [10], and eukaryotes [11]. For the global detection of genomic features, several RNA-seq-based protocols have been created. For example, differential RNA-seq (dRNA-seq) [12,13] represents a method for the system-wide mapping of transcriptional start sites (TSSs). For the construction of dRNA-seq libraries, a sample is split into two aliquots: one is digested by terminator exonuclease (the TEX+ library), which degrades processed RNA molecules with 5΄-monophosphate, while the other aliquot remains untreated (TEX- library). Both subsamples are then used to generate cDNA libraries. Based on this method, primary transcripts that have a 5΄-triphosphate are enriched in the TEX+ libraries. The digestion of matured transcript in the TEX+ library leads to a relative enrichment of primary transcripts. Thus, TSSs can be identified by comparing normalized coverage values between the TEX+ and TEX- libraries [12,13]. In addition to dRNA-seq, other RNA-seq-based protocols such as Term-seq [14] and ribosome profiling [15,16] have been applied to globally detect terminators, ORFs, and riboswitches but require dedicated data processing. While there are tools that can process RNA-seq data in order to predict genome-wide features such as TSSs based on dRNA-seq data [17,18,19] or based on conventional RNA-seq data [20,21], there has been,to date, no solution that combines different predictions of genomic features and compiles them into a consistent annotation.

Here we present ANNOgesic, a modular, command-line tool that can integrate data from different RNA-seq protocols such as dRNA-seq as well as conventional RNA-seq performed after transcript fragmentation and generate high-quality genome annotations that include features missing in most bacterial annotations (e.g., small noncoding RNAs, untranslated regions [UTRs], TSSs, and operons). The central approach is to detect transcript boundaries and then subsequently attach additional information about type as well as function to the predicted features and also to infer interactions between them. Several of ANNOgesic’s core functions represent new implementations that are not found in other programs. Third-party tools embedded into ANNOgesic are accessible via a consistent command-line interface. Furthermore, their results are improved, e.g., by dynamic parameter optimizations or by removing false positives. Numerous visualizations and statistics help the user to quickly evaluate the feature predictions. The tool is modular and has been intensively tested with several RNA-seq datasets from bacterial as well as from archaeal species.

## Materials and Methods

### Modules of ANNOgesic

ANNOgesic consists of the following modules, their names indicate their functions: Sequence modification, Annotation transfer, SNP/Mutation, Transcript, TSS, Terminator, UTR, Processing site (PS), Promoter, Operon, sRNA, sRNA target, small ORF (sORF), Gene Ontology (GO) term, Protein-protein interaction network, Subcellular localization, Riboswitch, RNA thermometer, Circular RNA, and Clustered regularly interspaced short palindromic repeat (CRISPR). Several potential workflows connecting these modules are displayed in Supplementary Fig. S1. An overview of the novelties and improvements of the modules in ANNOgesic are listed in Supplementary Table S1, and all the dependencies of ANNOgesic are shown in Supplementary Table S2.

Depending on the task, ANNOgesic requires a specific set of input information, either as coverage information in wiggle format or alignments in binary alignment map (BAM) format. This can be generated by short-read aligners such as STAR [22], segemehl [23], or a full RNA-seq pipeline such as READemption [24]. Certain modules additionally require annotations in GFF3 format. In case a sufficient genome annotation is not available, ANNOgesic can perform an annotation transfer from a closely related strain based on fasta and GFF3 files provided by the user.

### Implementation and installation

ANNOgesic’s source code is implemented in Python 3 and hosted at https://pypi.org/project/ANNOgesic/. The comprehensive documentation can be found at http://annogesic.readthedocs.io/, and releases are automatically submitted to Zenodo (https://zenodo.org/) to guarantee long-term availability. It can be easily installed using pip (https://pip.pypa.io). In order to provide a frictionless installation including non-Python dependencies, we additionally offer a Docker image at (https://hub.docker.com/r/silasysh/annogesic/) [25].

### Optimization of the parameter set for TSSpredator

**Figure 1:**
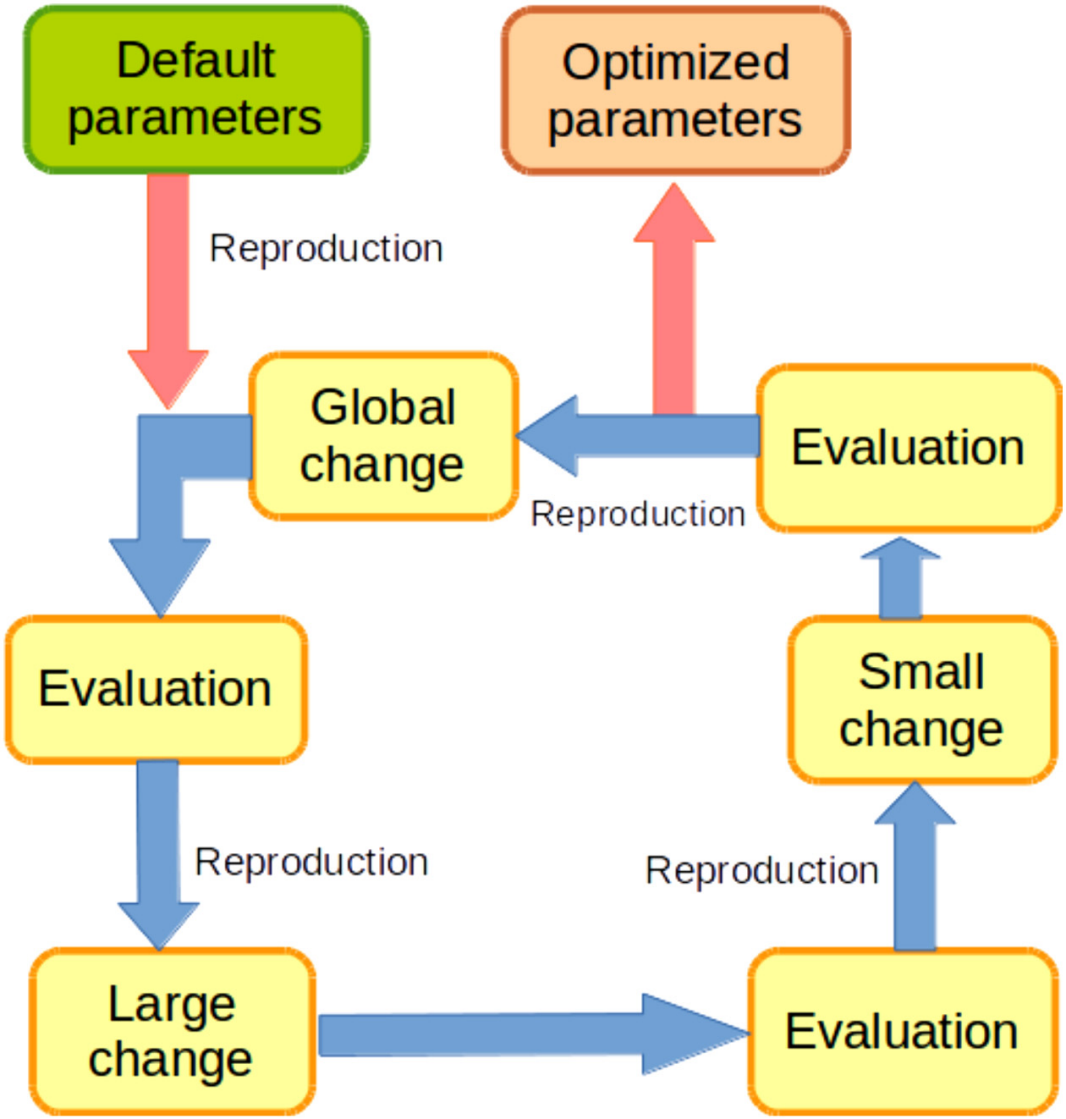
Schema of the genetic algorithm for optimizing the parameters of TSSpredator. It starts from the default parameters. These parameter sets will go through three steps: global change (change every parameter randomly), large change (change two of the parameters randomly), and then small change (adds/subtracts a small fraction to one of the parameters). It will then select the best parameter set for reproduction when one step is done. Usually, ANNOgesic can achieve the optimized parameters within 4,000 runs.

For several parts of ANNOgesic, the selection of parameters has a strong impact on the final results. Especially the TSS prediction, building on TSSpredator [17], requires a sophisticated fine-tuning of several parameters (namely, height, height reduction, factor, factor reduction, enrichment factor, processing site factor, and base height). To overcome the hard task of manual parameter selection, ANNOgesic optimizes the parameters by applying a genetic algorithm, a machine learning approach, [26] that is trained based on a small user-curated set of TSS predictions. This approach has the advantage of being able to find global, not only local, optima. The process of optimization is composed of three parts: random change, large change, and small change (Fig. 1). In this context, a global change means a random allocation of values to all parameters; a large change is a random allocation of values to two parameters; and a small change is adding or subtracting a small fraction to or from one parameter value. The result of each iteration is evaluated by a decision statement (Equation 1).

(1) \begin{eqnarray*}
TPR_{c}-TPR_{b} \ge 0.1 
\end{eqnarray*}

(2) \begin{eqnarray*}
(TPR_{c}\gt TPR_{b}) \wedge (FPR_{c}\lt FPR_{b}) 
\end{eqnarray*}

(3) \begin{eqnarray*}
(TP_{b}-TP_{c}\gt 0) \wedge (FP_{b}-FP_{c} \ge 5 \times (TP_{b}-TP_{c})) 
\end{eqnarray*}

(4) \begin{eqnarray*}
(TP_{b}-TP_{c}\lt 0) \wedge (FP_{c}-FP_{b} \le 5 \times (TP_{c}-TP_{b})) 
\end{eqnarray*}

(5) \begin{eqnarray*}
(TP_{m} \ge 100) \wedge (TPR_{c}-TPR_{b} \ge 0.01) \wedge (FPR_{c}-FPR_{b} \le 5 \times 10^{-5})
\end{eqnarray*}

(6) \begin{eqnarray*}
(TP_{m} \ge 100) \wedge (TPR_{b}-TPR_{c} \le 0.01) \wedge (FPR_{b}-FPR_{c} \ge 5 \times 10^{-5})
\end{eqnarray*}

In Equation 1, *TP*_*m*_ is the number of manually detected TSSs. *TP*_*c*_/*TPR*_*c*_ represents the true positive/true positive rate of the current parameters. *TP*_*b*_/*TPR*_*b*_ represents the true positive/true positive rate of the best parameters. *FP*_*c*_/*FPR*_*c*_ represents the false- positive/false-positive rate of the current parameters. *FP*_*b*_/*FPR*_*b*_ represents the false- positive/false-positive rate of the best parameters. If one of these six situations is true, it will replace the best parameters with current parameters.

### Test datasets

In order to test ANNOgesic’s performance, we applied it to RNA-seq datasets originating from *Helicobacter pylori* 26695 [7,13] and *Campylobacter jejuni* 81116 [17]. The dRNA-seq datasets were retrieved from (National Center for Biotechnology Information (NCBI) GEO where they are stored under the accession numbers GSE67564 and GSE38883, respectively. For *H. pylori* conventional RNA-seq data, i.e., without TEX treatment (which degrades transcripts without a 5’-triphosphate) and with fragmentation of the transcript before the library preparation, was also retrieved from NCBI SRA (accession number SRR031126). Moreover, for assessing the performance of ANNOgesic, dRNA-seq, and conventional RNA-seq datasets of *Escherichia coli*, K-12 MG1655 were downloaded from NCBI GEO (accession numbers GSE55199 and GSE45443; only the data of the wild-type strain were retrieved) [21,27]. The ANNOgesic predictions generated using these datasets of *E. coli* K-12 MG1655 were compared to the databases RegulonDB, EcoCyc, and DOOR^2^ [28 –33]

## Results

### Correction of genome sequences and annotations

All genomic features that can be detected by ANNOgesic are listed in Table 1. In order to demonstrate and test ANNOgesic’s performance, we analyzed RNA-seq data of *H. pylori* 26695 and *C. jejuni* 81116 and discuss the prediction results as examples in the following sections.

**Table 1:** Overview of feature predictions for *H. pylori* 26695 and *C. jejuni* 81116

		*H. pylori* 26695	*C. jejuni* 81116
Gene		1560	1685
Coding sequence (CDS)	Total	1448	1630
	Expressed	1406	1513
Transcript		1716	1147
TSS	Total	2458	1242
	Primary	703	565
	Secondary	156	92
	Internal	719	360
	Antisense	1161	510
	Orphan	111	30
Processing site		281	345
Terminator	Total	820, (437)	874, (375)
	TransTermHP	631, (314)	655, (269)
	Convergent genes	229, (151)	276, (145)
UTR	5’ UTR	693	560
	3’ UTR	325	286
sRNA	Total	184	40
	Intergenic	60	16
	Antisense	85	21
	5’ UTR-derived	10	0
	3’ UTR-derived	23	2
	InterCDS-derived	6	1
Operon	Total	554	710
	Monocistronic	268	386
	Polycistronic	286	324
sORF		150	25
Riboswitch		3	5
RNA thermometer		1	1
circular RNA		0	1
CRISPR		0	1, (8)

The numbers in parentheses for terminator and CRISPR represent occurrences of terminators with coverage drop and repeat units of CRISPR, respectively. For the prediction of terminators, ANNOgesic only keeps the high confidence ones in case a coding sequence (CDS) is associated with multiple terminators.

#### Genome sequence improvement and single nucleotide polymorphism/mutation calling

Conventionally, differences in the genome sequence of a strain of interest and the reference strain are determined by DNA sequencing. However, RNA-seq reads can also be repurposed to detect such single nucleotide polymorphisms (SNPs) or mutations that occur in transcribed regions, which can help to save the resources required for dedicated DNA sequencing or DNA SNP microarray measurements. The two drawbacks of this method are that only locations that are expressed can be analyzed and that, due to RNA editing, changes could be present only in the RNA level and are not found in the genome. On the other hand, it has been shown to be a valid approach for eukaryotic species and that the majority of SNPs are found in the expressed transcripts [34,35]. Such analysis could be useful to generate hypotheses that then need to be tested with complementary methods. ANNOgesic can perform SNPs/mutation calling via SAMtools [36] and BCFtools [36] applying read counting-based filtering.

#### Annotation transfer

ANNOgesic integrates the Rapid Annotation Transfer Tool [37], which can detect the shared synteny and mutations between a reference and query genome to transfer annotation (i.e., genes, CDSs, tRNAs, rRNAs) by applying MUMmer [38]. For the chosen strains, *H. pylori* 26695 and *C. jejuni* 81116 annotation files in GFF3 format were obtained from NCBI RefSeq. Because of this, there was no need to transfer the annotation from a closely related strain.

### Detection of transcripts

**Figure 2:**
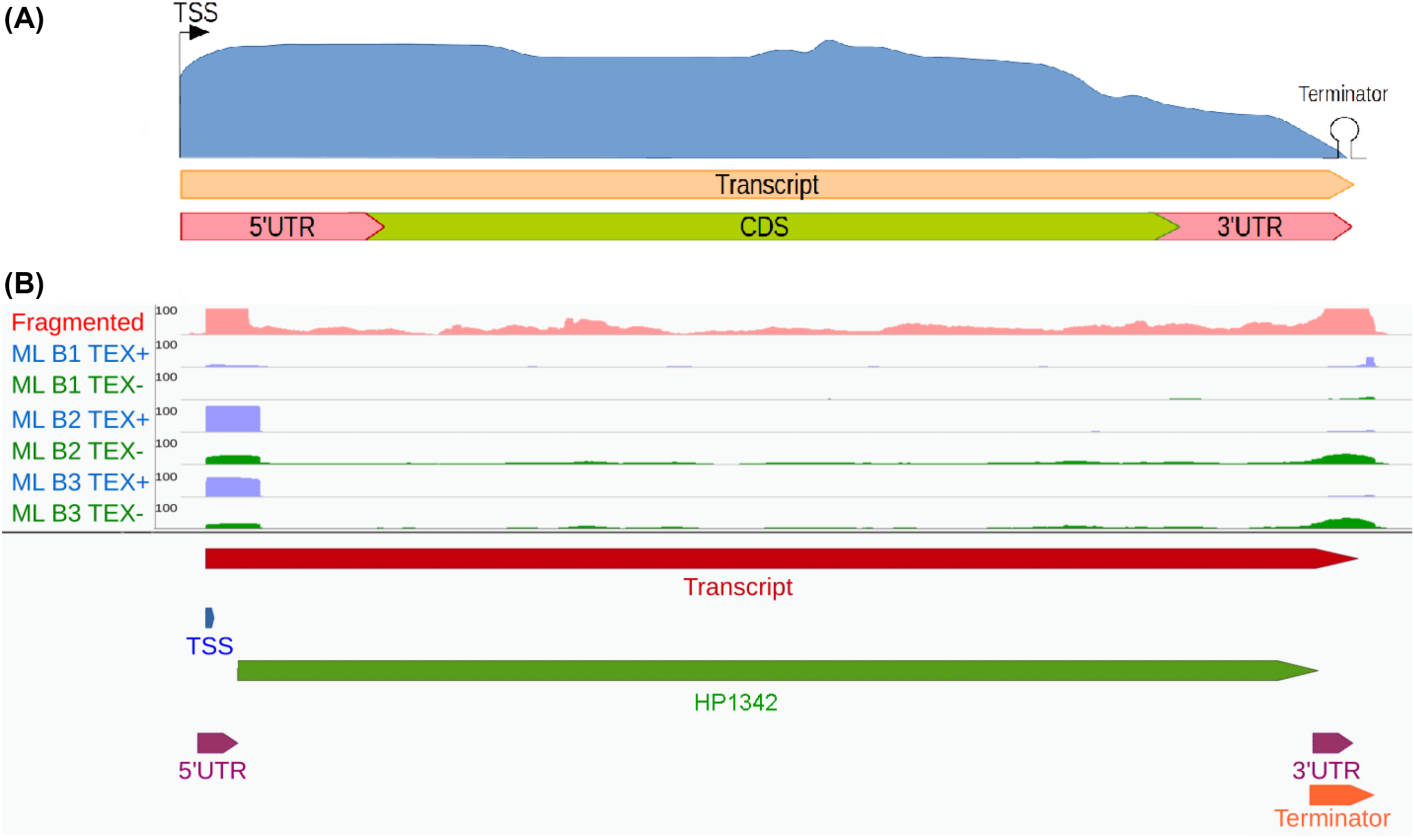
Transcript boundary detection. **(A)** Schema: ANNOgesic can predict TSSs, terminators, transcripts, genes, and UTRs and integrate them into a comprehensive annotation. **(B)** Gene HP1342 of *H. pylori* 26695 as an example. The pink coverage plot represents RNA-seq data of libraries after fragmentation, the blue coverage plots TEX+ libraries of dRNA-seq, and the green coverage plots TEX- libraries of dRNA-seq. Transcript, TSS, terminator, and CDS are presented as red, blue, orange, and green bars, respectively. The figure shows how the transcript covers the whole gene location and how UTRs (presented by purple bars) can be detected based on the TSS, transcript, terminator, and gene annotations.

Knowing the exact boundaries and sequence of a transcript is crucial for a comprehensive understanding of its behavior and function. For example, UTRs can be the target of regulation by sRNAs or small molecules (e.g., riboswitches) [39,40] or even sources of sRNAs [41]. Unfortunately, most bacterial annotations only cover the protein-coding regions, while the information about TSSs, terminators, and UTRs is lacking. To address this issue, ANNOgesic combines several feature predictions for a reliable detection of transcripts and their boundaries (Fig. 2).

#### Coverage-based transcript detection

**Figure 3:**
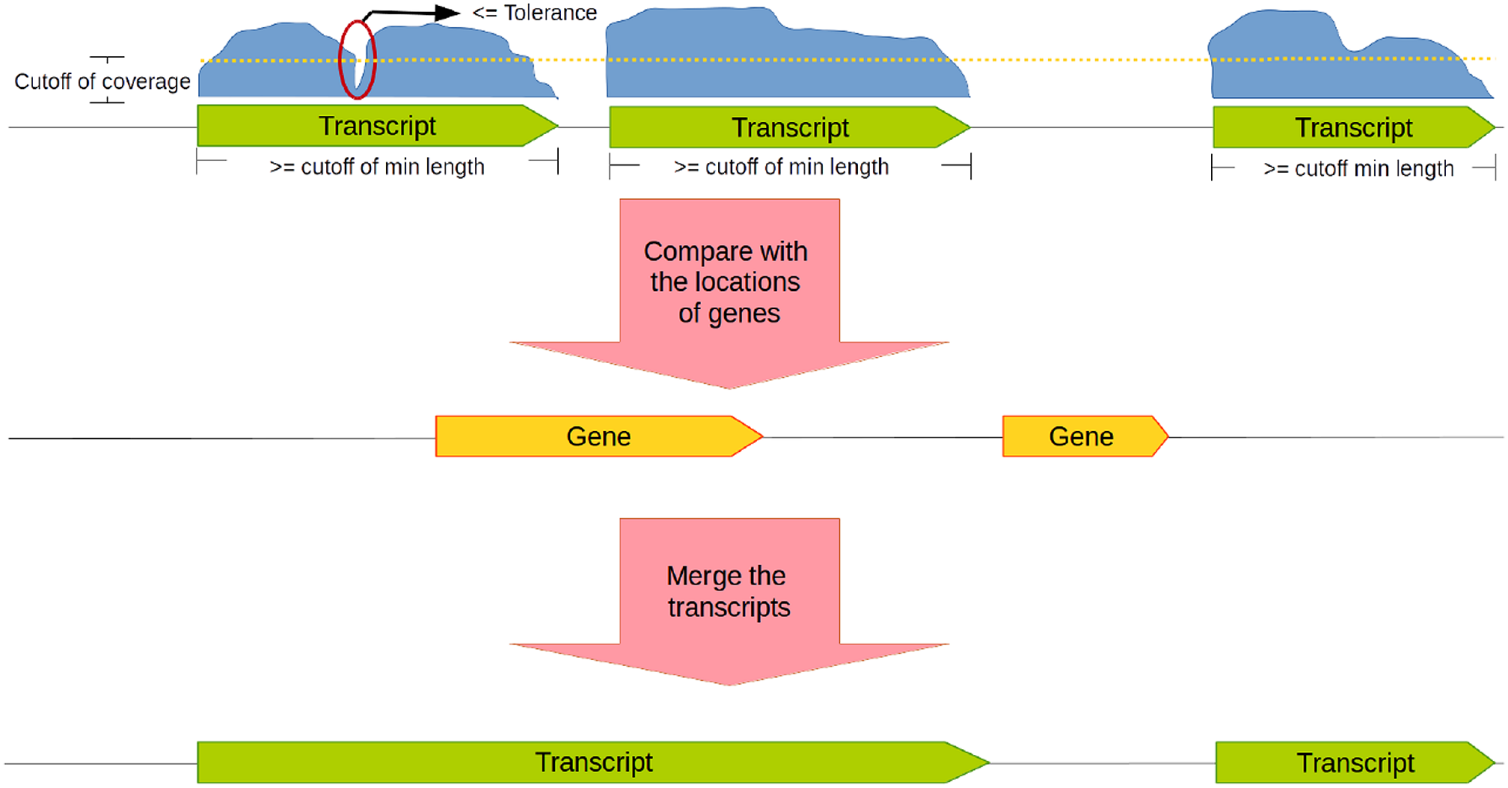
Coverage-based transcript detection. If the coverage (blue curve-blocks) is higher than a given coverage cutoff value (dashed line), a transcript will be called. The user can set a tolerance value (i.e., a number of nucleotides with a coverage below the cutoff) on which basis gapped transcripts are merged or are kept separated. Information of gene positions can also be used to merge transcripts in case two of them overlap with the same gene.

There are numerous tools available for the detection of transcripts (e.g., [42]), but most of them are optimized for the assembly of eukaryotic transcripts. Because of this, we combined several heuristics to perform such predictions. Nucleotide coverage data are used for defining the expressed regions, and genome annotations are applied for extending or merging the gene expressed regions to form complete transcripts. Several parameters such as the threshold of coverage values can be set by the user to fine-tune the predictions (Fig. 3).

By running ANNOgesic’s subcommand for transcript prediction, we detected 1,716 transcripts in *H. pylori* 26695 and 1,147 transcripts in *C. jejuni* 81116. These transcripts cover 1,520 and 1,568 genes, which shows that 97% and 93% of the known genes are expressed in at least one condition, respectively.

#### Transcriptional start sites

For the prediction of TSSs, ANNOgesic builds on TSSpredator [17], which takes dRNA-seq coverage data as input. The outcome of TSSpredator’s predictions depends strongly on the setting of numerous parameters, and fine-tuning those can be time consuming. Because of this, a parameter optimization was implemented in ANNOgesic that builds on a small, manually curated set of TSSs to find optimal values.

In order to test the performance of ANNOgesic, we manually annotated TSSs in the first 200 kb of the genome of *H. pylori* 26695 and *C. jejuni* 81116 (Supplementary Tables S5 and S6). This set was used to perform the predictions of TSSpredator with default settings as well with the parameters optimized by ANNOgesic. For the test set of the benchmarking, we manually annotated TSSs from first 200 kb to 400 kb in the genome of *H. pylori* 26695 and *C. jejuni* 81116 (Supplementary Tables S5 and S6). As displayed in Table 2, the optimization had minor sensitivity improvements in *H. pylori* 26695 (from 96.8% to 99.6%); it strongly increased the sensitivity for the TSS prediction for *C. jejuni* 81116 (67.1% to 98.7%) while keeping the same level of specificity. To underpin those findings, we looked at the overlap of the predicted TSS and predicted transcripts. This was nearly the same for *H. pylori* 26695 (82% for default and 83% for optimized setting) but also increased significantly for *C. jejuni* 81116 from 81% for default parameters to 96% with optimized parameters.

**Table 2:** Comparison of default and optimized parameters of TSSpredator for TSS and PS prediction

Strain	Parameter	Sensitivity (TP)	Specificity (FP)
TSS			
*H. pylori* 26695	Default	96.8% (244)	99.98% (32)
	Optimization	99.6% (251)	99.98% (32)
*C. jejuni* 81116	Default	67.1% (104)	99.98% (31)
	Optimization	98.7% (153)	99.99% (7)
PS			
*H. pylori* 26695	Default	92.9% (26)	99.99% (7)
	Optimization	92.9% (26)	99.99% (7)
*C. jejuni* 81116	Default	61.3% (19)	99.99% (2)
	Optimization	93.5% (29)	99.99% (6)

The numbers in parentheses represent true positive or false positive.

Moreover, TSSs are classified depending on their relative positions to genes by TSSpredator. Based on these classifications, Venn diagrams representing the different TSS classes are automatically generated (Supplementary Fig. S2).

#### Processing sites

Several transcripts undergo processing, which influences their biological activity [41,43]. In order to detect PSs based on dRNA-seq data, ANNOgesic facilitates the same approach as described for TSS detection but searches for the reverse enrichment pattern, i.e., a relative enrichment in the library not treated with TEX in comparison to the library treated with TEX. This coverage pattern is observed as the TEX enzyme will not degrate processed transcripts due to the missing triphosphate at the 5’end, which leads to a relative enrichment in samples. As done for the TSSs, we manually annotated the PSs in the first 200 kb of the genomes by looking for such enrichment patterns. Based on these manually curated sets, we performed parameter optimization on the test set (manually curated from the first 200 kb to 400 kb; Supplementary Tables S7 and S8, Table 2) and could improve the prediction of PSs by TSSpredator [17]. With optimized parameters 281 and 345, PSs were detected in *H. pylori* 26695 and *C. jejuni* 81116, respectively.

#### ρ-independent terminators

While the TSSs are in general clearly defined borders, the 3’-end of a transcript is often not very sharp. A commonly used tool for the prediction of the 3’-end of a transcript is TransTermHP [44], which detects ρ-independent terminators based on genome sequences. Manual inspection showed us that TransTermHP predictions are not always supported by the RNA-data (Supplementary Fig. S3E and S3F). This could be due to the lack of expression in the chosen conditions. Additionally, certain locations in 3’-ends that may be ρ-independent were not detected by TransTermHP. Because of this, we extended the prediction by two additional approaches based on RNA-seq coverage and the given genome sequence. At first, terminators predicted by TransTermHP that show a significant decrease of coverage are marked as high-confidence terminators. For this, the drop of coverage inside the predicted terminator region plus 30 nucleotides upstream and downstream is considered sufficient if the ratio of the lowest coverage value and the highest coverage value is at a user-defined value (the default value is 0.5, and the schemes and examples are shown in Supplementary Fig. S3). In order to improve the sensitivity, an additional heuristic for the detection of ρ-independent terminators was developed. In this approach, only converging gene pairs (i.e., the 3’-end are facing each other) are taken into account (Supplementary Fig. S4). In case the region between the two genes is A/T-rich and a stem-loop can be predicted in there, the existence of a ρ-independent terminator is assumed. As a default, the region should consist of 80 or fewer nucleotides, the T-rich region should contain more than 5 thymines, the stem-loop needs to be 4-20 nucleotides, the length of the loop needs to be between 3 and 10 nucleotides, and a maximum of 25% of the nucleotides in the stem should be unpaired.

#### UTRs

Based on the CDS locations and the above-described detection of TSSs, terminators, and transcripts, 5’ UTR and 3’ UTR can be annotated by ANNOgesic. Additionally, it visualizes the distribution of UTR lengths in a histogram (as shown in Supplementary Fig. S5).

#### Promoters

ANNOgesic integrates MEME [45], which detects ungapped motifs, and GLAM2 [46], which discovers gapped motifs, for the detection and visualization of promoter motifs. The user can define the number of nucleotides upstream of TSSs that should be screened and the length of potential promoter motifs. The motifs can be generated globally or for the different types of TSSs (example in Supplementary Fig. S6).

#### Operons

**Figure 4:**
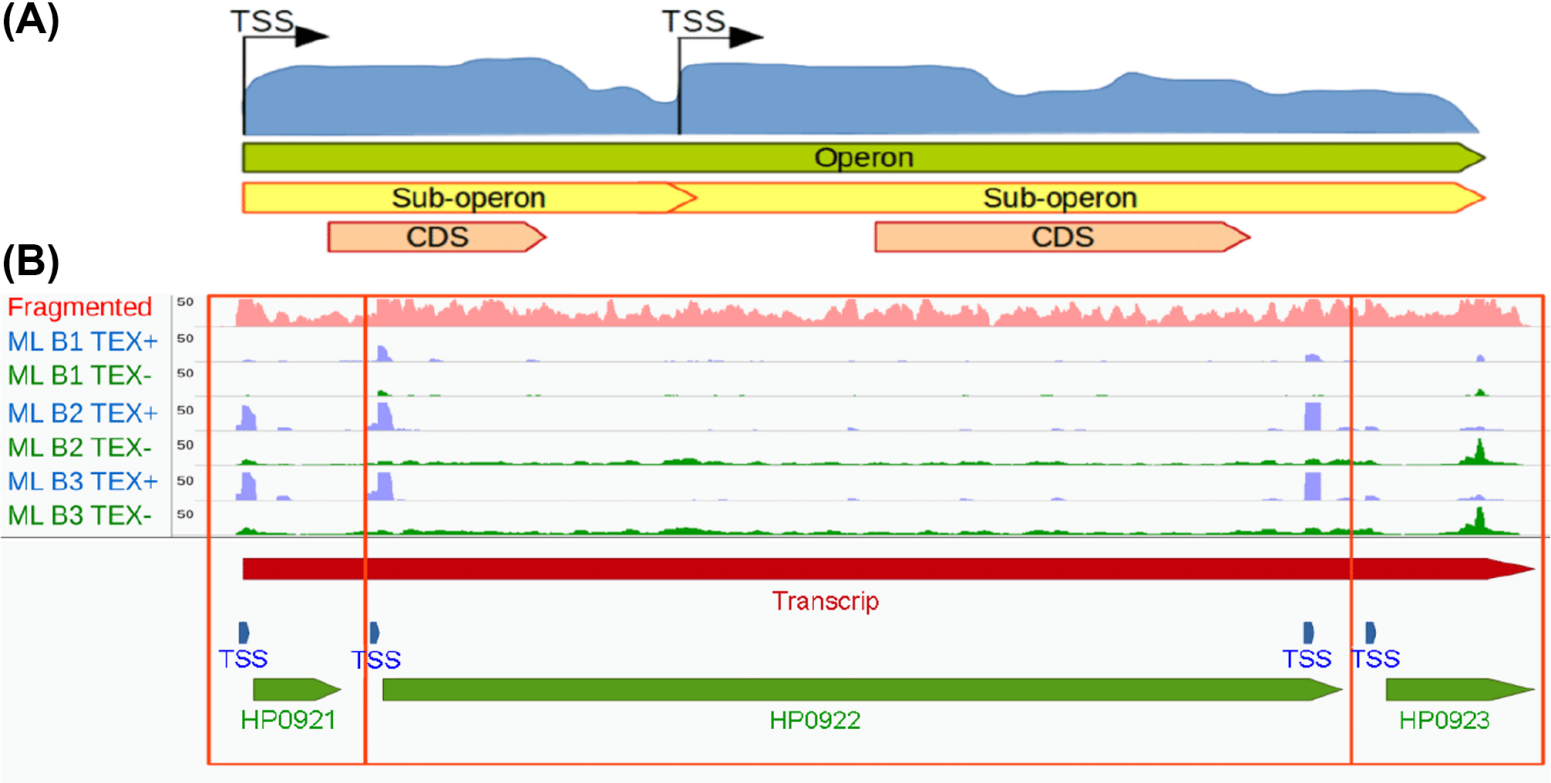
Operon and sub-operon detection. **(A)** If there is more than one TSSs that does not overlap with genes located within one operon, the operon can be divided to several sub-operons based on these TSSs. **(B)** An example from *H. pylori* 26695. The coverage of RNA-seq with fragmentation, TEX+, and TEX- of dRNA-seq are shown in pink, blue, and green coverages, respectively. TSSs, transcripts/operons, and genes are presented as blue, red, and green bars, respectively. The two genes are located in the same operon but also in different sub-operons (two empty red squares).

Based on the TSS and transcript prediction, ANNOgesic can generate statements regarding the organization of genes in operons and suboperons as well as report the number of monocistronic operons and polycistronic operons (Fig. 4).

### Detection of sRNAs and their targets

**Figure 5:**
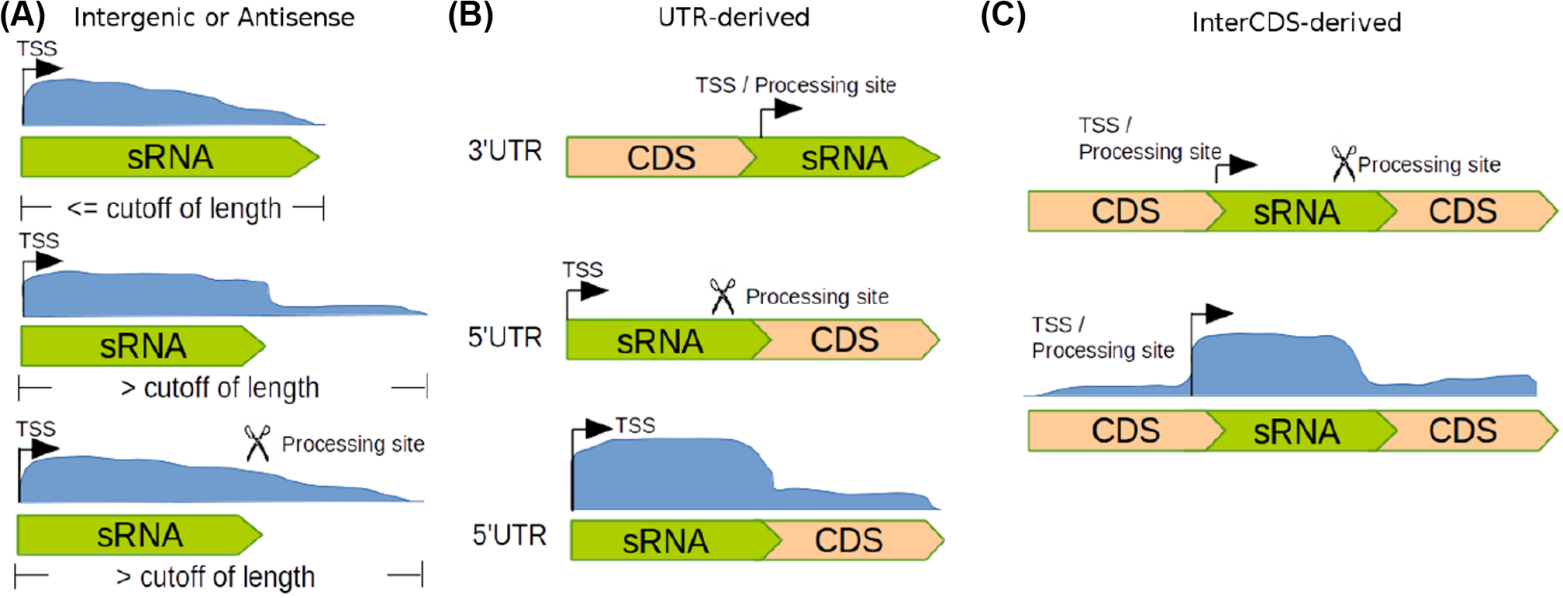
Detection of intergenic, antisense, and UTR-derived sRNAs. The length of potential sRNAs should be within a given range, and their coverages should exceed a given minimum coverage. **(A)** Detection of intergenic and antisense sRNAs. Three potential cases are shown. In the upper panel, the transcript starts with a TSS, and length of the transcript is within the expected length. In the middle panel, the transcript starts with a TSS, but the transcript is longer than an average sRNA. In that case, ANNOgesic will search in the region of high coverage (blue region) for a point at which the coverage is decreasing rapidly. In the bottom panel, the image is identical to the one in the middle, but the sRNA ends instead with a PS. **(B)** Detection of UTR-derived sRNAs. For 3’ UTR-derived sRNAs: if the transcript starts with a TSS or PS, it will be tagged as a 3’ UTR-derived sRNA. For 5’ UTR-derived sRNAs: if the transcript starts with a TSS and ends with a PS or the point where the coverage significant drops. **(C)** Detection of interCDS-derived sRNAs; this is similar to the 5’ UTR-derived approach, but the transcript starts with a PS.

The detection of sRNAs based on RNA-seq data is a nontrivial task. While numerous sRNAs are found in intergenic regions, several cases of 5’ and 3’ UTR-derived sRNAs are reported [41,47, 48,^49^]. ANNOgesic offers the detection of all classes combined with a detailed characterization of the sRNA candidates (Fig. 5).

In order to classify newly detected intergenic transcripts as sRNAs, ANNOgesic tests several of their features. If a Basic Local Alignment Search Tool + [50] search of a transcript finds homologous sequences in BSRD [51], a database that stores experimentally confirmed sRNAs, the transcript gets the status of an sRNA. The user can also choose additional databases for searching homologous sequences. In case a search against the NCBI nonredundant protein database leads to a hit, it is marked as potentially protein-coding. Otherwise, a transcript must have a predicted TSS, form a stable secondary structure (i.e., the folding energy change calculated with RNAfold from Vienna RNA package [52] must be below a user-defined value), and their length should be in the range of 30 to 500 nt in order to be tagged as an sRNA. All these requirements are used per default but can be modified or removed via ANNOgesic’s command line parameters. ANNOgesic stores the results of all analyses and generates GFF3 files, fasta files, secondary structural figures, dot plots, as well as mountain plots based on those predictions.

For sRNAs that share a transcript with CDSs—5’ UTR, inter-CDS, or 3’ UTR located sRNAs—we implemented several detection heuristics (Fig. 5B and 5C). The 5’ UTR-derived sRNAs must start with a TSS and show a sharp drop of coverage or a PS in the 3’-end. The requirement for the detection of inter-CDS located sRNAs is either a TSS or a PS as well as a coverage drop at the 3’-end or a PS. Small RNAs derived from the 3’ UTR are expected to have a TSS or a PS and either end with the transcript or at a PS. After the detection of a *bona fide* sRNA, the above-described quality filters (e.g., length range, secondary structure) are applied to judge the potential of a candidate (examples are shown in Supplementary Figs. S7, S8). For the validation of sRNA candidates in our test case, the described sRNAs of two publications were chosen. Sharma et al. [7] described 63 sRNAs of which 4 were not expressed in the condition of the test dataset (removed from the dataset) (Supplementary Fig. S9). Of these 59, 53 (90%) were detected by ANNOgesic. In the *C. jejuni* 81116 set, 31 sRNAs were described by Dugar et al. [17], and ANNOgesic could recover 26 (84%). The sRNA ranking system provided by ANNOgesic is displayed in Supplementary Fig. S10 and Supplementary Equation S1.

In order to deduce potential regulatory functions of newly predicted sRNAs, ANNOgesic performs prediction of interaction between them and mRNAs using RNAplex [52,53], RNAup [52,54], and IntaRNA [55]. The user can choose if only interactions supported by all tools are reported.

### Detection of sORFs

**Figure 6:**
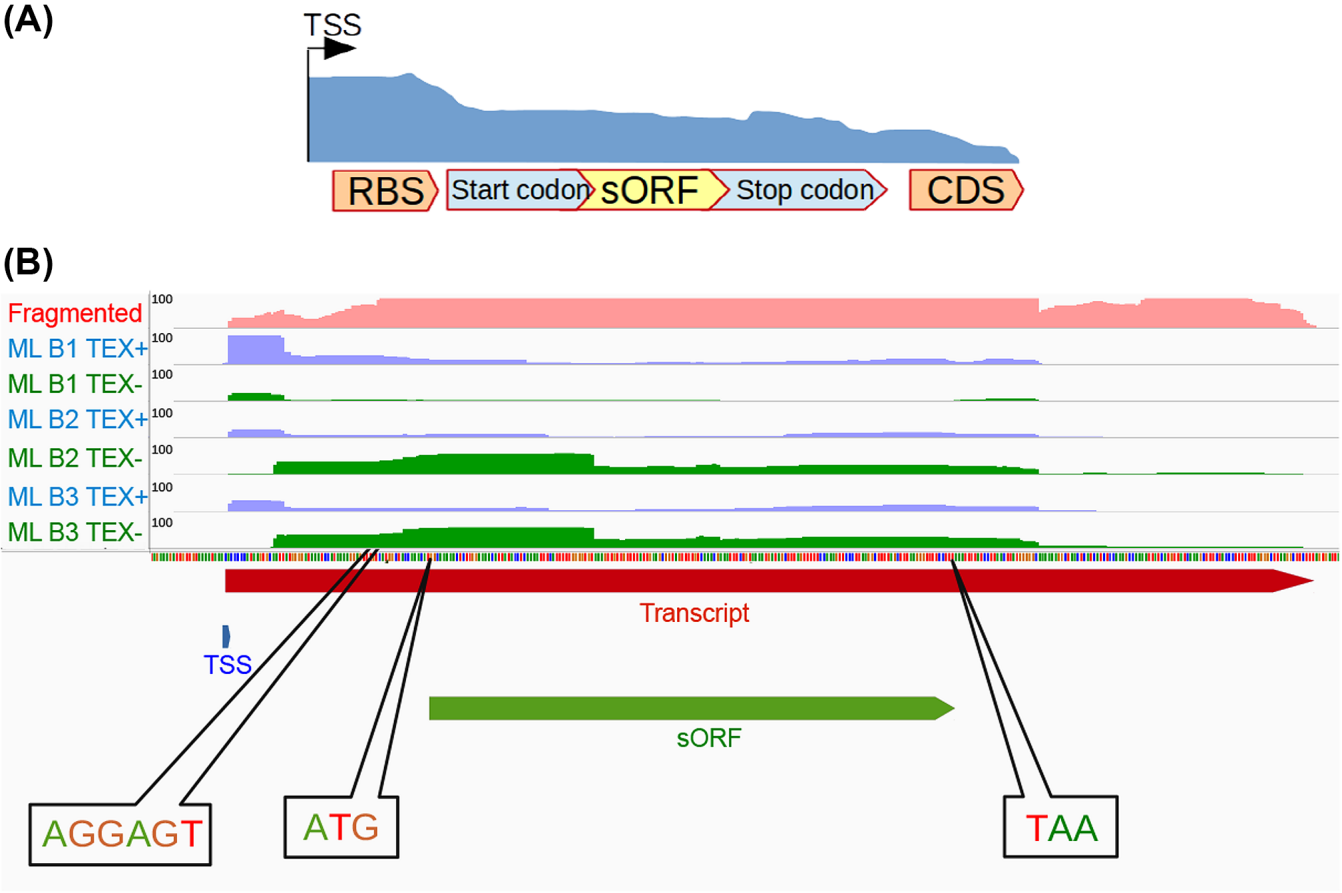
sORF detection. **(A)** An sORF must contain a start codon and stop codon within a transcript and should be inside of a given length range (default 30 -150nt). Additionally, a ribosomal binding site must be detected between the TSS and the start codon. **(B)** An example from *H. pylori* 26695. The coverage of RNA-seq (fragmented libraries), TEX+, and TEX- (dRNA-seq) are shown as pink, blue, and green coverages, respectively. The TSS, transcript, and sORF are presented as blue, red, and green bars, respectively.

All newly detected transcripts that do not contain a previously described CDS as well all 5’ UTRs and 3’ UTRs are scanned for potential sORFs [56] (Fig. 6). For this, ANNOgesic searches for start and stop codons (noncanonical start codons are not included but can be assigned by the user) that constitute potential ORFs of 30 to 150 base-pairs. Furthermore, ribosomal binding sites (based on the Shine-Dalgarno sequence, but different sequences can be assigned as well) between the TSS and 3 to 15 bp upstream of the start codon are required for a *bona fide* sORF.

### Detection of functional-related attributes

In order to facilitate a better understanding of the biological function of known and newly detected transcripts, ANNOgesic predicts several attributes for these features.

This includes the allocation of GO as well as GOslim [57] terms to CDSs via searching of protein ids in Uniprot [58]. The occurrence of groups is visualized for expressed and nonexpressed CDSs (Supplementary Fig. S11). Furthermore, the subcellular localization is predicted by PSORTb [59] for the proteins (Supplementary Fig. S12). Additionally, the protein entries are enriched by protein-protein interaction information retrieved from STRING [60] and PIE [61] (examples in Supplementary Fig. S13).

### Circular RNAs

ANNOgesic integrates the tool ”testrealign.x” from the segemehl package for the detection of circular RNAs [62] and adds a filter to reduce the number of false positive. Candidates for circular RNAs must be located in intergenic regions and exceed a given number of reads.

### CRISPRs

CRISPR/Cas systems represent a bacterial defense system against phages and consist of repeat units and spacer sequences as well as Cas proteins [63]. The CRISPR Recognition Tool [64] is integrated into ANNOgesic and extended by comparison of CRISPR/Cas candidates to other annotations to remove false positive (Supplementary Fig. S14).

### Riboswitches and RNA thermometers

Riboswitches and RNA thermometers are regulatory sequences that are part of transcripts and influence the translation based on the concentration of selected small molecules and temperature change, respectively. For the prediction of these riboswitches and RNA thermometers, ANNOgesic searches [65] the sequences that are between TSSs (or starting point of a transcript if no TSS was detected) and downstream CDSs, as well as those associated with ribosome binding site in the Rfam database using Infernal [66].

### Comparison between ANNOgesic predictions and published databases for *E. coli* K-12

In order to assess the performance of ANNOgesic, we compared its predictions based on a dRNA-seq dataset and conventional RNA-seq of *E. coli* K-12 MG1655 by Thomason et al. [27] and McClure et al. [21] with the entries in several databases [28–33]. Most of the benchmarking features can be precisely detected (80% or more) (Supplementary Table S3). Moreover, the predicted features not found in published databases have the high possibility to be novel features that are strongly supported by RNA-seq data (Supplementary Fig. S7B, S7D). TSSs represent an exception with lower success rates, and we assume this is mostly due to the higher sensitivity of the dRNA-seq method in comparison to older protocols. To test this assumption and to investigate the quality of the TSS entries in RegulonDB, we compared the three deposited TSS datasets (Salgado et al. generated with Illumina RNA-seq as well as Mendoza-Vargas et al. generated with Roche 454 high-throughput pyrosequencing and generated with Roche 5’RACE [67,68]) to each other and found very small overlap (Supplementary Fig. S15). Additionally, the 50 nucleotides upstream of TSSs were extracted and scanned with MEME [45] for common motifs that are similar to the ones described for promoters. Only for a small number, 0% to 7%, of TSSs such motifs were found (Supplementary Table S4), while 80% of the TSS predictions from ANNOgesic have such a promoter motif located upstream (Supplementary Figure S6C). The same analysis could not be performed with EcoCyc [28], which is lacking TSS information and provides only positions but no strand information for promoters. Because of these results, we doubt that the data in those databases represent a solid ground for benchmarking the accuracy of ANNOgesic’s TSS predictions.

## Discussion

While RNA-seq has become a powerful method for annotating genomes, the integration of its data is usually very laborious and time consuming. It requires bioinformatic expertise and involves the application of different programs to perform the different required steps. Here, we present ANNOgesic, a modular, user-friendly annotation tool for the analysis of bacterial RNA-seq data that integrates several tools, optimizes their parameters, and includes novel prediction methods for several genomic features. With the help of this command-line tool, RNA-seq data can be efficiently used to generate high-resolution annotations of bacterial genomes with very little manual effort. In addition to the annotation files in standard formats, it also returns numerous statistics and visualizations that help the user to explore and to evaluate the results. While it ideally integrates conventional RNA-seq (beneficial for detecting 3’-ends of transcripts) as well as dRNA-seq (required for the efficient detection of internal TSSs) as input together (see Supplementary Figs. S16 and S17), it can also perform sufficient predictions with only one class of data for the majority of the genomic features (Supplementary Table S3).

Here, we demonstrated the performance of ANNOgesic by applying it on two published datasets and comparing the results to manually conducted annotations. ANNOgesic could detect 90% and 83% of the manually annotated sRNAs *H. pylori* 26695 and *C. jejuni* 81116, respectively. The sRNAs missed by ANNOgesic can be explained by low coverage, not being associated with TSSs or lack of expression in the assayed conditions (see Supplementary Figs. S18 and S19).

In addition to the analyses presented as examples in this study (*H. pylori* 26695 and *C. jejuni* 81116), ANNOgesic was successfully applied for detecting transcripts, sRNAs, and TSSs in additional annotation projects (e.g., *Pseudomonas aeruginosa* [69] and *Rhodobacter sphaeroides* [70]). Despite the fact that the program was developed mainly with a focus on bacterial genomes, it has also been used to annotate archaeal genomes (namely *Methanosarcina mazei* [Lutz et al., unpublished]) and eukaryotic genomes that have no introns (*Trypanosoma brucei* [Müller et al., unpublished]).

ANNOgesic is freely available under the OSI-compliant ISCL open source license (some of the dependencies are available under other FLOSS licenses), and extensive documentation has been produced to guide novice and advanced users.

## Conclusions

ANNOgesic is a powerful tool for annotating genome features based on RNA-seq data from multiple protocols. ANNOgesic not only integrates several available tools but also improves their performance by optimizing parameters and removing false positives. For the genomic features that cannot be detected using available tools, several novel methods have been developed and implemented as part of ANNOgesic. Comprehensive documentation and useful statistics as well as visualizations are also provided by ANNOgesic.

## Availability of supporting source code and requirements

- Project name: ANNNOgesic
- Project home page: GitHub - https://github.com/Sung-Huan/ANNOgesic.
  PyPI - https://pypi.org/project/ANNOgesic/.
  DockerHub - https://hub.docker.com/r/silasysh/annogesic/.
- SciCrunch RRID: SCR_016326
- Operating system(s): Linux, Mac OS
- Programming language: Python
- Other requirements: Please check the documentation (http://annogesic.readthedocs.io/en/latest/required.html).
- License: ISC (Internet Systems Consortium license, simplified BSD license).

## Supplementary Material

GIGA-D-18-00029-Original_Submission.pdf

GIGA-D-18-00029_Revision_1.pdf

GIGA-D-18-00029_Revision_2.pdf

Response_to_Reviewer_Comments_Original_Submission.pdf

Response_to_Reviewer_Comments_Revision1.pdf

Reviewer_1_Report_(Original_Submission) -- A. Rebecca Wattam 02-26-2018 Reviewed

Reviewer_2_Report_(Original_Submission).pdf -- Raquel Tobes, Ph.D 04-14-2018 Reviewed

Reviewer_3_Report_(Original_Submission).pdf -- Roy Chaudhuri 07-17-2018 Reviewed

Reviewer_3_Report_Revision_1.pdf -- Roy Chaudhuri 07-06-2018 Reviewed

Supplement_Files.zip

## Availability of supporting data

Snapshots of the code and data are available in the GigaScience repository, GigaDB [71]. Code and data are also available via the Code Ocean reproducibility platform [72]. For information on the supporting files, please check the documentation (http://annogesic.readthedocs.io/en/latest/subcommands.html).

## Additional files

**Figure S1:** Workflow charts of ANNogesic modules.

**Figure S2:** Distribution of TSS classes.

**Figure S3:** Concept and examples for detecting coverage decrease of terminators.

**Figure S4:** Terminator prediction approach based on convergent genes.

**Figure S5:** Length distribution of UTRs.

**Figure S6:** The promoter motifs detected in *Helicobacter pylori* 26695, *Campylobacter jejuni* 81116, and *Escherichia coli* K-12 MG1655.

**Figure S7:** Examples of known and novel intergenic sRNAs that ANNOgesic can detect.

**Figure S8:** Examples of detected antisense and UTR derived sRNAs.

**Figure S9:** The coverage plots of the benchmarking sRNA HPnc4620.

**Figure S10:** Histograms of ranking number of the sRNA benchmarking set.

**Figure S11:** The distributions of GO term.

**Figure S12:** The distributions of subcellular localizations.

**Figure S13:** Visualization of protein-protein interactions.

**Figure S14:** The example of CRISPR in *Campylobacter jejuni* 81116.

**Figure S15:** The overlap of three previously published TSS datasets in RegulonDB.

**Figure S16:** The predicted sRNA which can be detected only in data RNA-seq after transcript fragmentation.

**Figure S17:** The comparison between dRNA-seq and RNA-seq after transcript fragmentation for detecting transcript

**Figure S18:** The lowly expressed sRNA - HPnc4610.

**Figure S19:** An example of known sRNA – CJnc230 – which is not associated with a TSS.

**Equation S1:** The ranking system of sRNA prediction.

**Table S1:** The novelties and improvements of genomic feature detection in ANNOgesic.

**Table S2:** The dependencies of the modules of ANNOgesic.

**Table S3:** The comparison between ANNOgesic predictions and several databases.

**Table S4:** The number of TSSs and their associated promoter motifs in RegulonDB.

**Table S5:** The manually-curated TSS set of *Helicobacter pylori* 26695 (1-400bp).

**Table S6:** The manually-curated TSS set of *Campylobacter jejuni* 81116 (1-400bp).

**Table S7:** The manually-curated PS set of *Helicobacter pylori* 26695 (1-400bp).

**Table S8:** The manually-curated PS set of *Campylobacter jejuni* 81116 (1-400bp).

## Abbreviations

CDS: coding sequence; CRISPR: clustered regularly interspaced short palindromic repeat; dRNA-seq: differential RNA sequencing; GO: Gene Ontology; nt: nucleotide; PS: processing site; sORF: small open reading frame; SNP: single nucleotide polymorphism; TSS: transcriptional start site; UTR: untranslated region.
