# Supplementary material for: ANNOgesic: a Swiss army knife for the RNA-seq based annotation of bacterial/archaeal genomes
Source: Gigascience. 2018 Aug 30;7(9):giy096. doi: 10.1093/gigascience/giy096 (PMC6123526; doi:10.1093/gigascience/giy096)
Supplement: Supplement_Files.zip [file giy096_supplement_files.zip › ANNOgesic_supplementary.pdf]

ANNOgesic: A Swiss army knife for the RNA-Seq based annotation of bacterial/archaeal genomes

Sung-Huan Yu [1], Jörg Vogel [1], and Konrad U. Förstner [1,2*]

[1]Institute of Molecular Infection Biology (IMIB), University of Würzburg, 97080 Würzburg, Germany
[2]Core Unit Systems Medicine, University of Würzburg, 97070 Würzburg, Germany

To whom correspondence should be addressed. Tel: +49-931/31-84279 ; Email: konrad.foerstner@uni-wuerzburg.de

Supplementary Figures

A

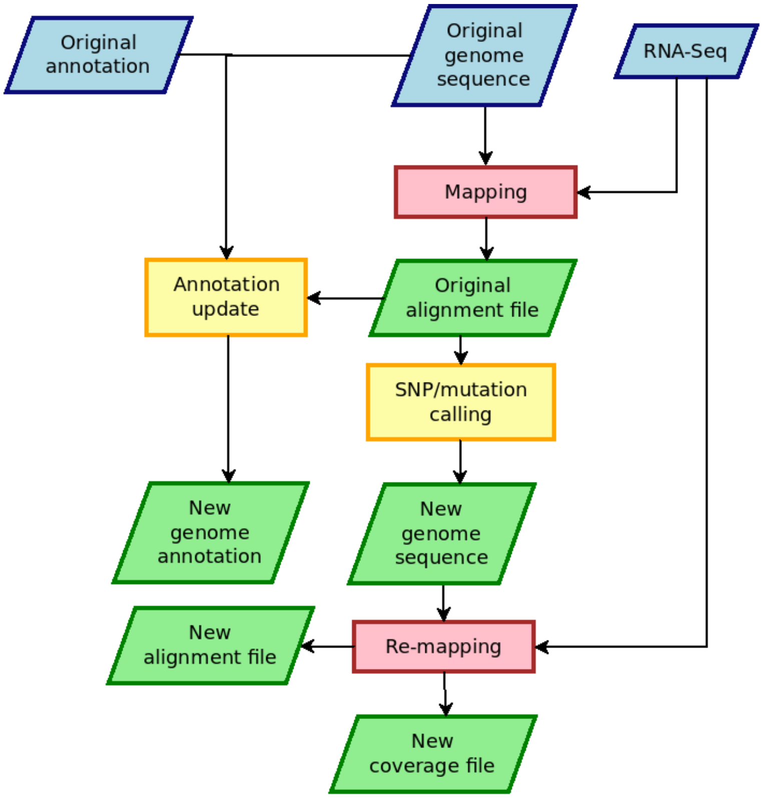

B

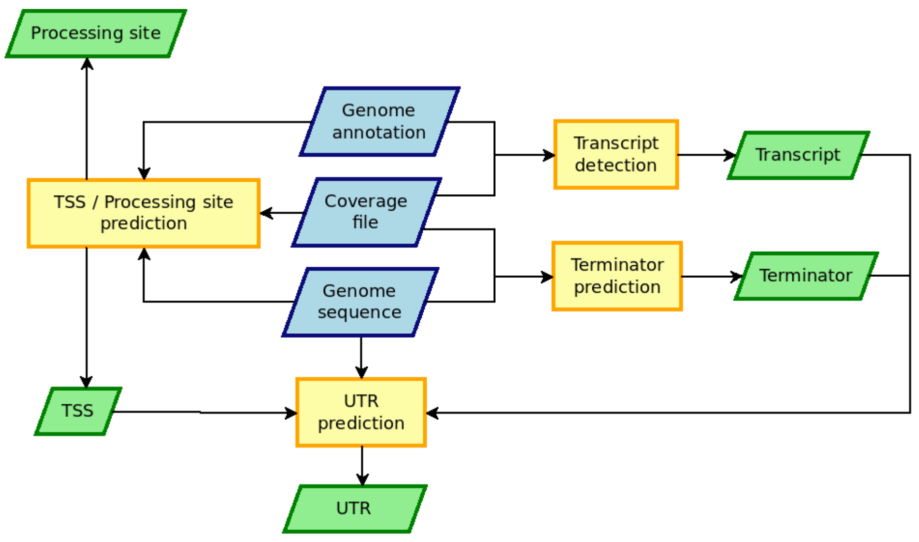

Processing site
Genome annotation
Transcript detection
Transcript
TSS / Processing site prediction
Coverage file
Terminator prediction
Terminator
Genome sequence
TSS
UTR prediction
UTR

C

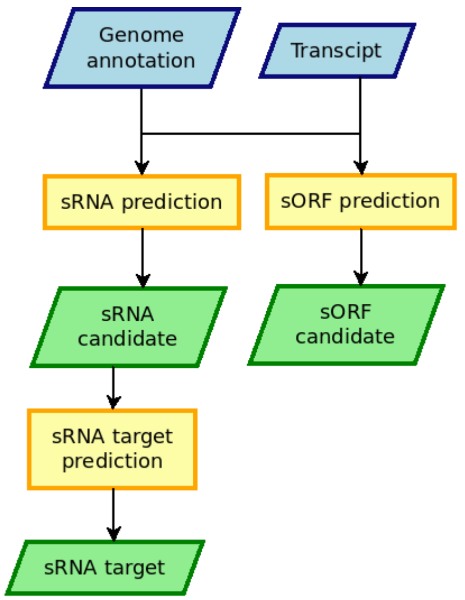

Genome annotation
Transcipt
sRNA prediction
sORF prediction
sRNA candidate
sORF candidate
sRNA target prediction
sRNA target

D

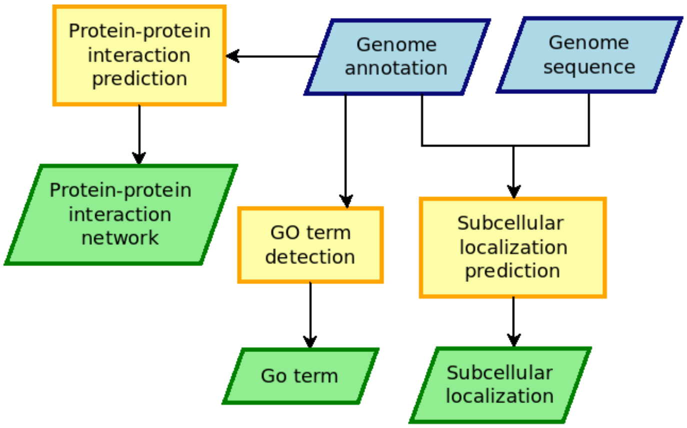
Protein-protein interaction prediction
Genome annotation
Genome sequence
Protein-protein interaction network
GO term detection
Subcellular localization prediction
Go term
Subcellular localization

E

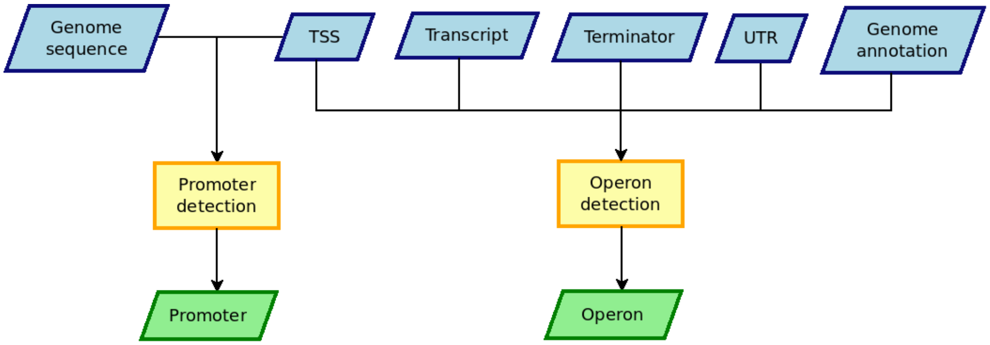
Genome sequence
TSS
Transcript
Terminator
UTR
Genome annotation
Promoter detection
Operon detection
Promoter
Operon

F

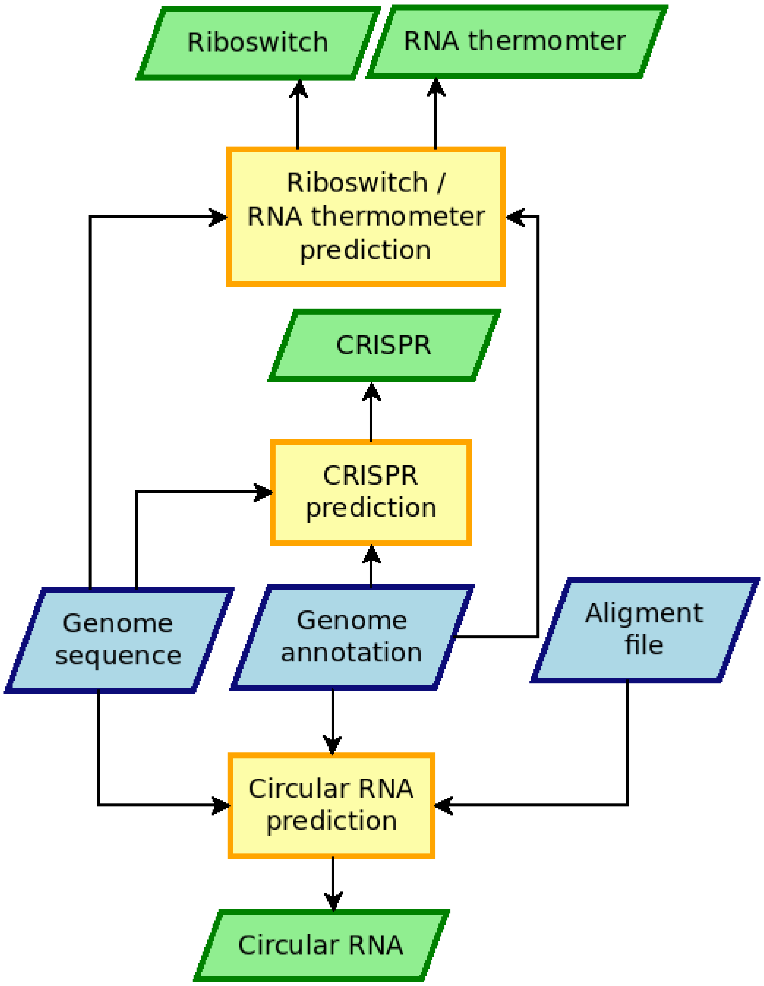

Figure S1: Workflow charts of ANNogesic modules. The yellow blocks represent the tools or methods of detection. The red blocks indicate that it is performed by the third-party tools. The blue parallelograms and the green parallelograms are input and output, respectively. **(A)** Reference genome improvement, **(B)** Transcript boundary, **(C)** Small RNA and small ORF, **(D)** Regulatory feature, **(E)** Promoter and Operon and **(F)** Other features.

A

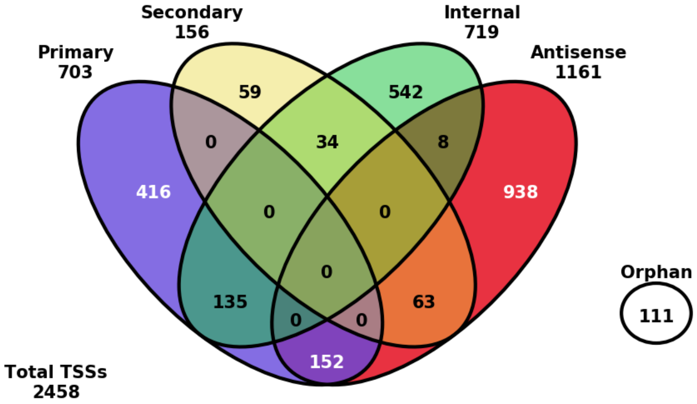

B

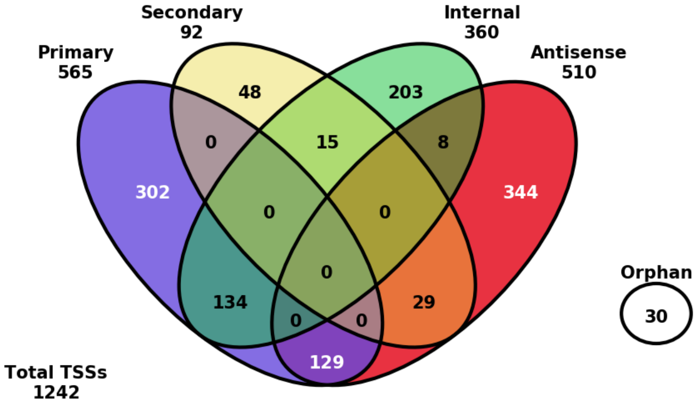

Figure S2: Distribution of TSS classes. **(A)** *Helicobacter pylori* 26695. **(B)** *Campylobacter jejuni* 81116.

A

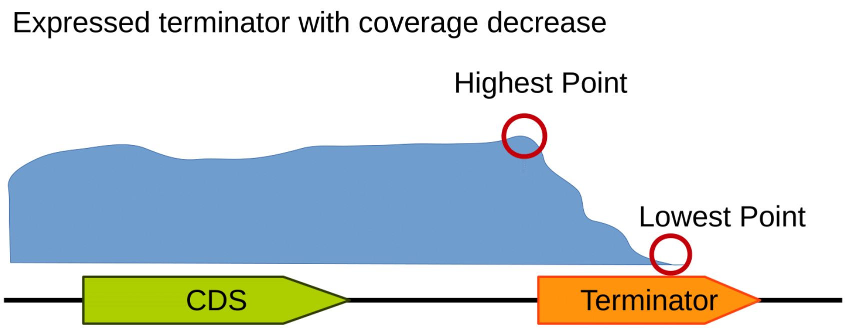

Expressed terminator with coverage decrease
Highest Point
Lowest Point
CDS
Terminator

B

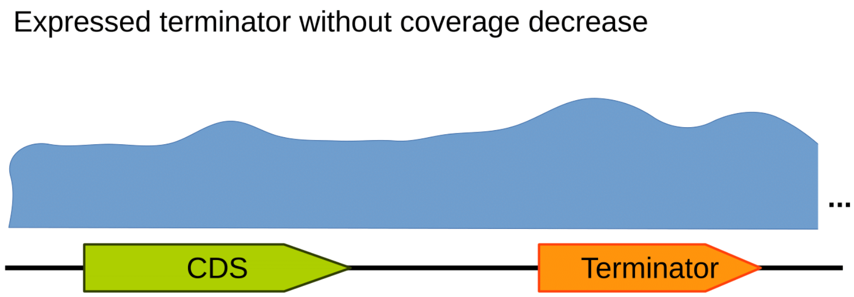

Expressed terminator without coverage decrease
...
CDS
Terminator

C

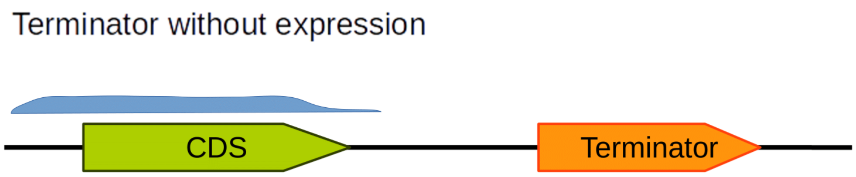

Terminator without expression
CDS
Terminator

D

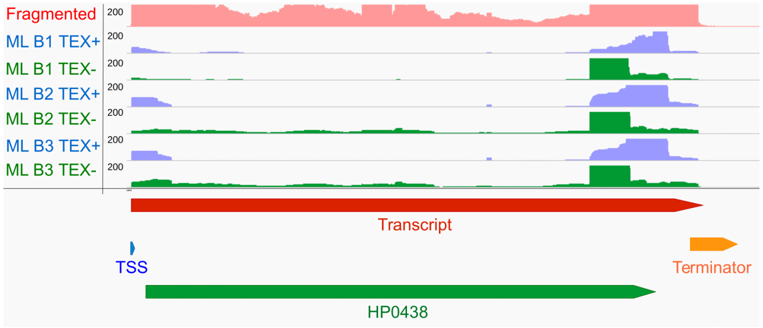

E

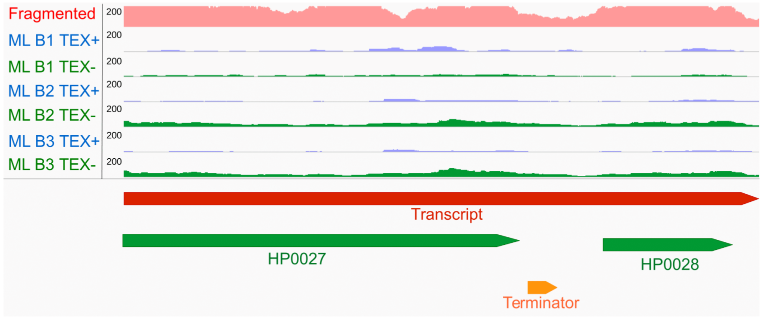

F

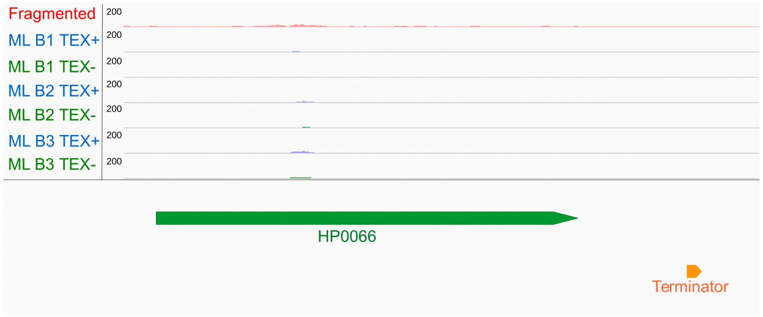

Figure S3: Concept and examples for detecting coverage decrease of terminators. (A) and (D) An expressed terminator with a significant coverage drop. The ratio of the lowest coverage value and the highest coverage values is lower than 0.5 (default). (B) and (E) An expressed terminator without coverage decrease. (C) and (F) A terminator without expression. In (D), (E), and (F), the coverage of RNA-Seq with transcript fragmentation, TEX+ and TEX- of dRNA-Seq are presented as pink, blue and green coverages, respectively. Terminators, TSSs, CDSs and transcripts are showed as orange, blue, green and red bars, respectively.

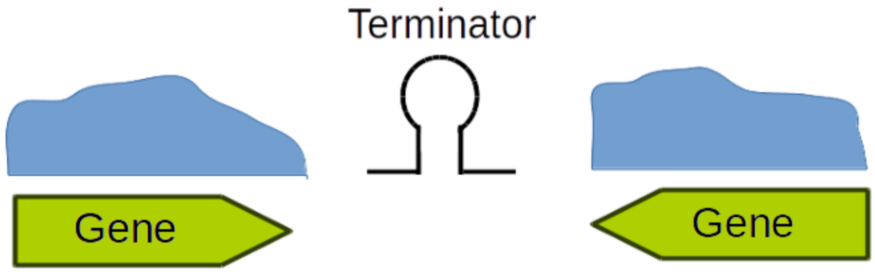

Figure S4: Terminator prediction approach based on convergent genes. The blue curve-blocks represent the coverages; the green arrows show two genes from different strands. Ideally, there should be a ρ-independent terminator within the region of two converging genes.

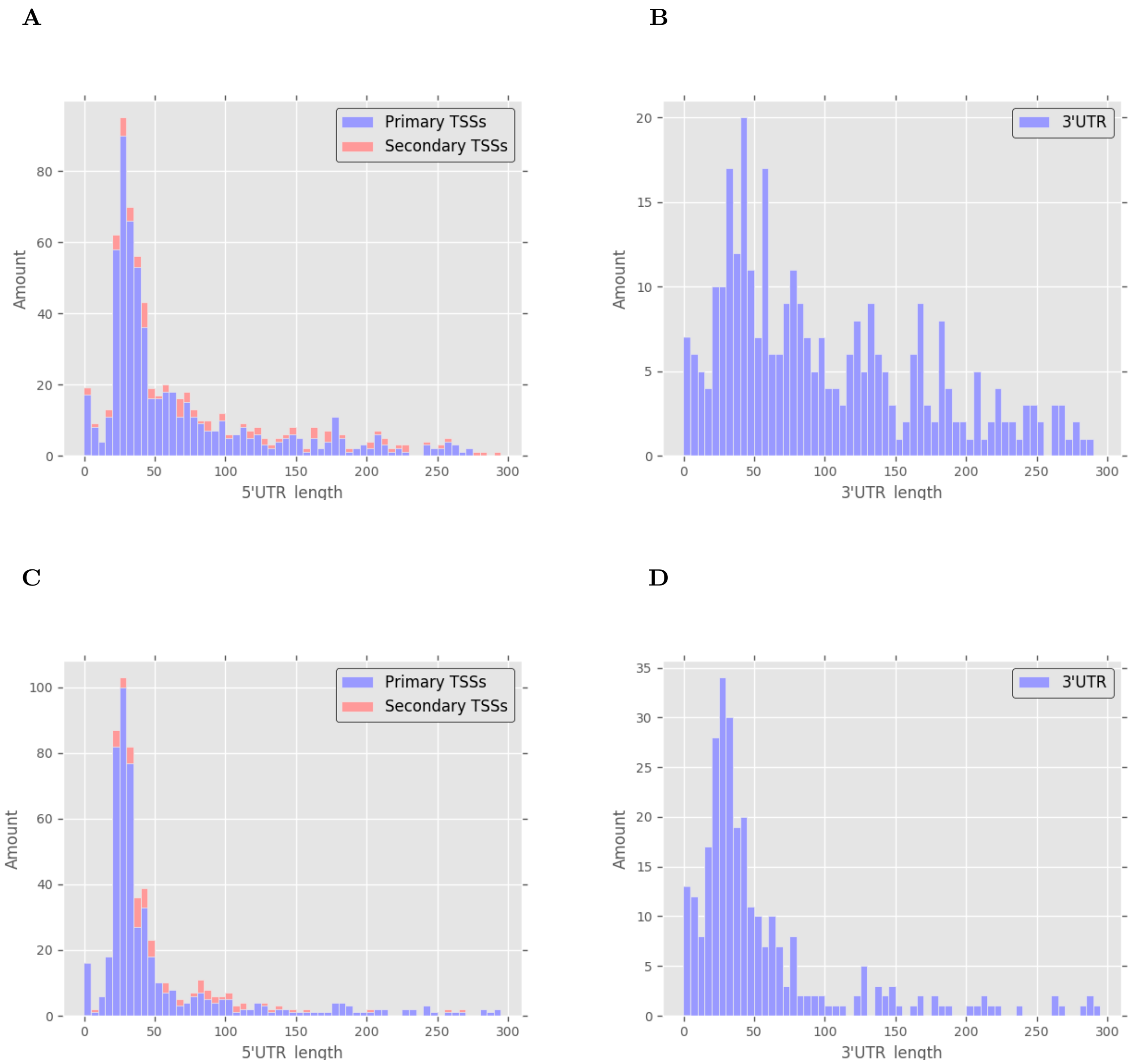

Figure S5: Length distribution of UTRs. For 5’ UTR the blue bars represent primary TSSs and the pink bars represent secondary TSSs. **(A)** 5’ UTRs of *Helicobacter pylori* 26695. **(B)** 3’ UTRs of *Helicobacter pylori* 26695. **(C)** 5’ UTRs of *Campylobacter jejuni* 81116. **(D)** 3’ UTRs of *Campylobacter jejuni* 81116.

A

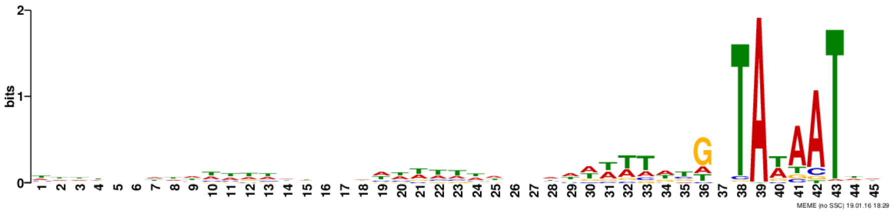

B

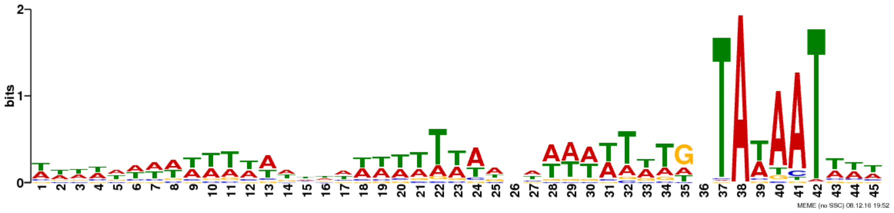

C

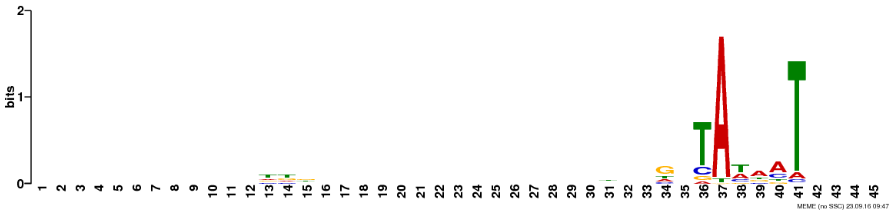

Figure S6: The promoter motifs detected in **(A)** *Helicobacter pylori* 26695 (found in front of 2297 TSSs i.e. 93.4%), **(B)** *Campylobacter jejuni* 81116 (associated with 1093 TSSs, 88%), and **(C)** *Escherichia coli* K-12 MG1655 (identified upstream of 11516 TSSs, 80%).

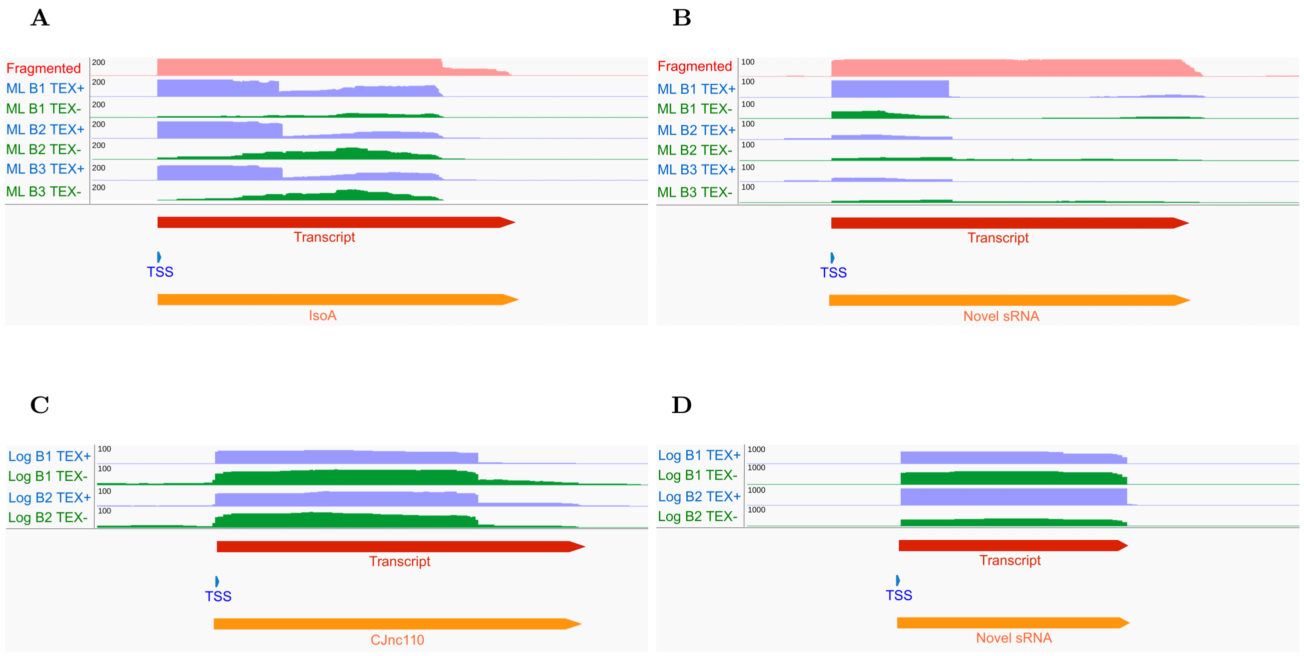

Figure S7: Examples of known and novel intergenic sRNAs that ANNOgesic can detect. The coverage of RNA-Seq with fragmentation, TEX+ and TEX- of dRNA-Seq are presented as pink, blue and green curves, respectively. In the annotation track sRNAs, TSSs, CDSs and transcripts are showed as orange, blue, green and red bars, respectively. **(A)** IsoA (HPnc7630) of *Helicobacter pylori* 26695 **(B)** Novel sRNA in *Helicobacter pylori* 26695 **(C)** CJnc110 in *Campylobacter jejuni* 81116 **(D)** novel sRNA in *Campylobacter jejuni* 81116.

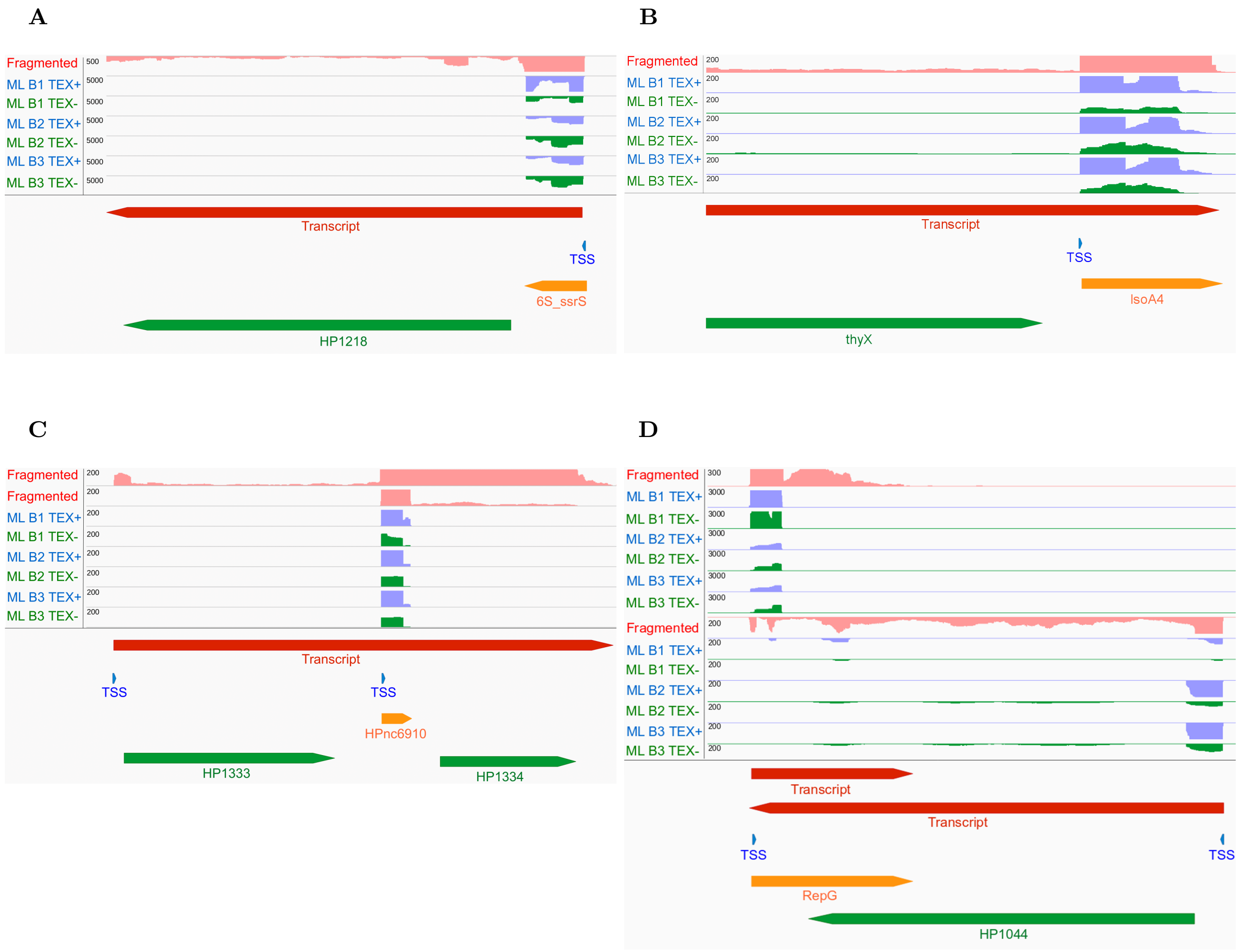

Figure S8: Examples of detected antisense and UTR derived sRNAs in *Helicobacter pylori* 26695. The coverage of RNA-Seq with fragmentation, TEX+ and TEX- of dRNA-Seq are presented as pink, blue and green coverages, respectively. In the annotation track sRNAs, TSSs, CDSs and transcripts are showed as orange, blue, green and red bars, respectively. **(A)** 5' UTR-derived sRNA – the sRNA and CDS are in the same transcript and the sRNA is located in the 5' UTR. **(B)** 3' UTR-derived sRNA – the sRNA and CDS are in the same transcript and the sRNA is located in 3' UTR. **(C)** InterCDS-derived sRNA – the sRNA and CDSs are in the same transcript, and the sRNA is located in the non-annotated region between two CDS. The two pink coverages are from the same fragmented library, but presented in different scales. **(D)** Antisense sRNA.

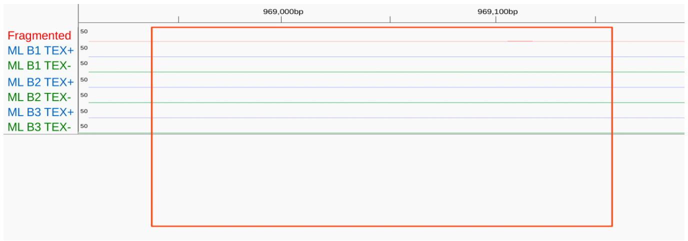

Figure S9: The coverage plots of the sRNA HPnc4620 which was excluded from the benchmarking set of *Helicobacter pylori* 26695. It is located in the region from base 968980 to 969164 (marked by the orange hollow square) of *Helicobacter pylori* 26695 and has no expression.

Ranking of sRNA

A simple heuristic was developed to ranks sRNA candidates by taking the presences of promoter into account (Supplementary Equation 1). In case a Pribnow box is detected in front of a sRNA, the score is the average coverag value multiplied by 2. If this is not the case, the score is simply the average coverage. The distribution of scores is shown in Supplementary Figure 10. Previously described sRNA show in general a high score. The p-values of a t-test between the list of benchmarking sets and the remaining ones are 1.631e-09 and 4.629e-04 for *Helicobacter pylori* and *Campylobacter jejuni* 81116, respectively. This indicates that the ranking system in ANNOgesic is a useful approach for selection of sRNAs for an experimental validation.

$$
\begin{aligned}
&if\ sRNA\ is\ associated\ with\ a\ promoter:\\
&\quad S = C \times P\\
&else:\\
&\quad S = C
\end{aligned}
$$

Equation S1: S is the score for ranking sRNAs. If the sRNA is not associated with a promoter, S is the average coverage of the sRNA (presented by C). If a promoter is found upstream of the sRNA, S is assigned by P times of the average coverage of the sRNA. P can be defined by the user.

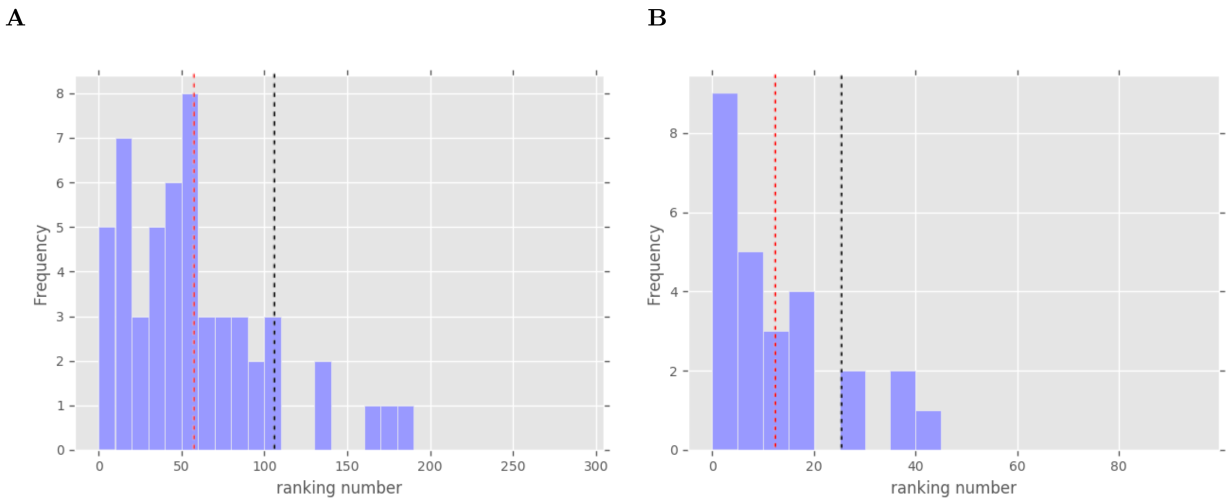

Figure S10: Histograms (**(A)** for *Helicobacter pylori* 26695 and **(B)** for *Campylobacter jejuni* 81116) of ranking number of the sRNA benchmarking set. The red dashed line represents the average ranking number of the benchmarking sets (57.25 and 13.19 of *Helicobacter pylori* 26695 and *Campylobacter jejuni* 81116, respectively), and the black dashed line shows the average ranking number of the remaining populations (106.17 and 25.05 of *Helicobacter pylori* 26695 and *Campylobacter jejuni* 81116, respectively).

A

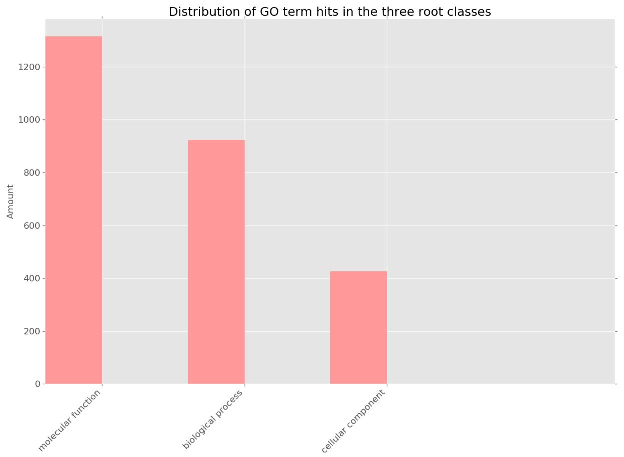
Distribution of GO term hits in the three root classes
Amount
molecular function
biological process
cellular component

B

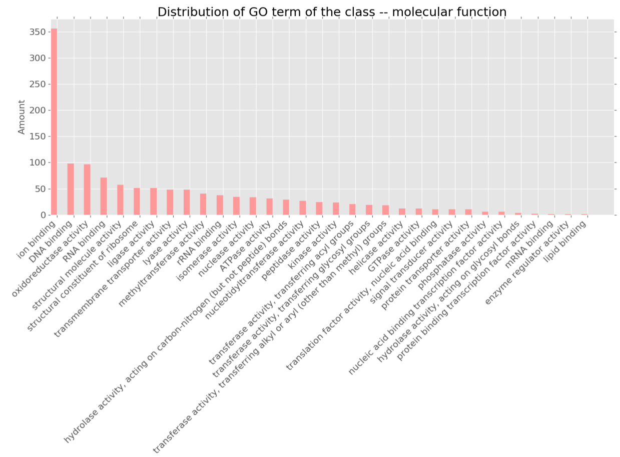
Distribution of GO term of the class -- molecular function
Amount
ion binding
DNA binding
oxidoreductase activity
RNA binding
structural molecule activity
structural constituent of ribosome
ligase activity
transmembrane transporter activity
lyase activity
methyltransferase activity
rRNA binding
isomerase activity
nuclease activity
ATPase activity
hydrolase activity, acting on carbon-nitrogen (but not peptide) bonds
nucleotidyltransferase activity
peptidase activity
kinase activity
transferase activity, transferring acyl groups
transferase activity, transferring glycosyl groups
transferase activity, transferring alkyl or aryl (other than methyl) groups
helicase activity
GTPase activity
translation factor activity, nucleic acid binding
signal transducer activity
protein transporter activity
phosphatase activity
nucleic acid binding transcription factor activity
hydrolase activity, acting on glycosyl bonds
protein binding transcription factor activity
mRNA binding
enzyme regulator activity
lipid binding

C

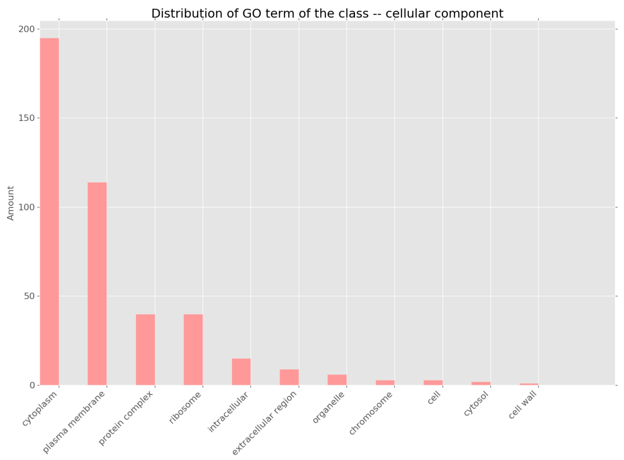
Distribution of GO term of the class -- cellular component
Amount
cytoplasm
plasma membrane
protein complex
ribosome
intracellular
extracellular region
organelle
chromosome
cell
cytosol
cell wall

D

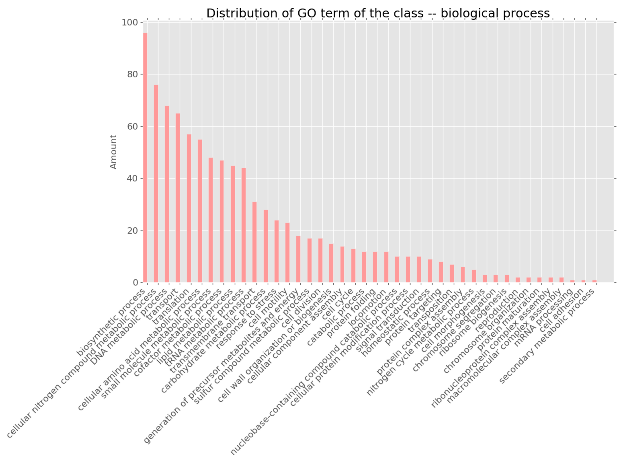
Distribution of GO term of the class -- biological process
Amount

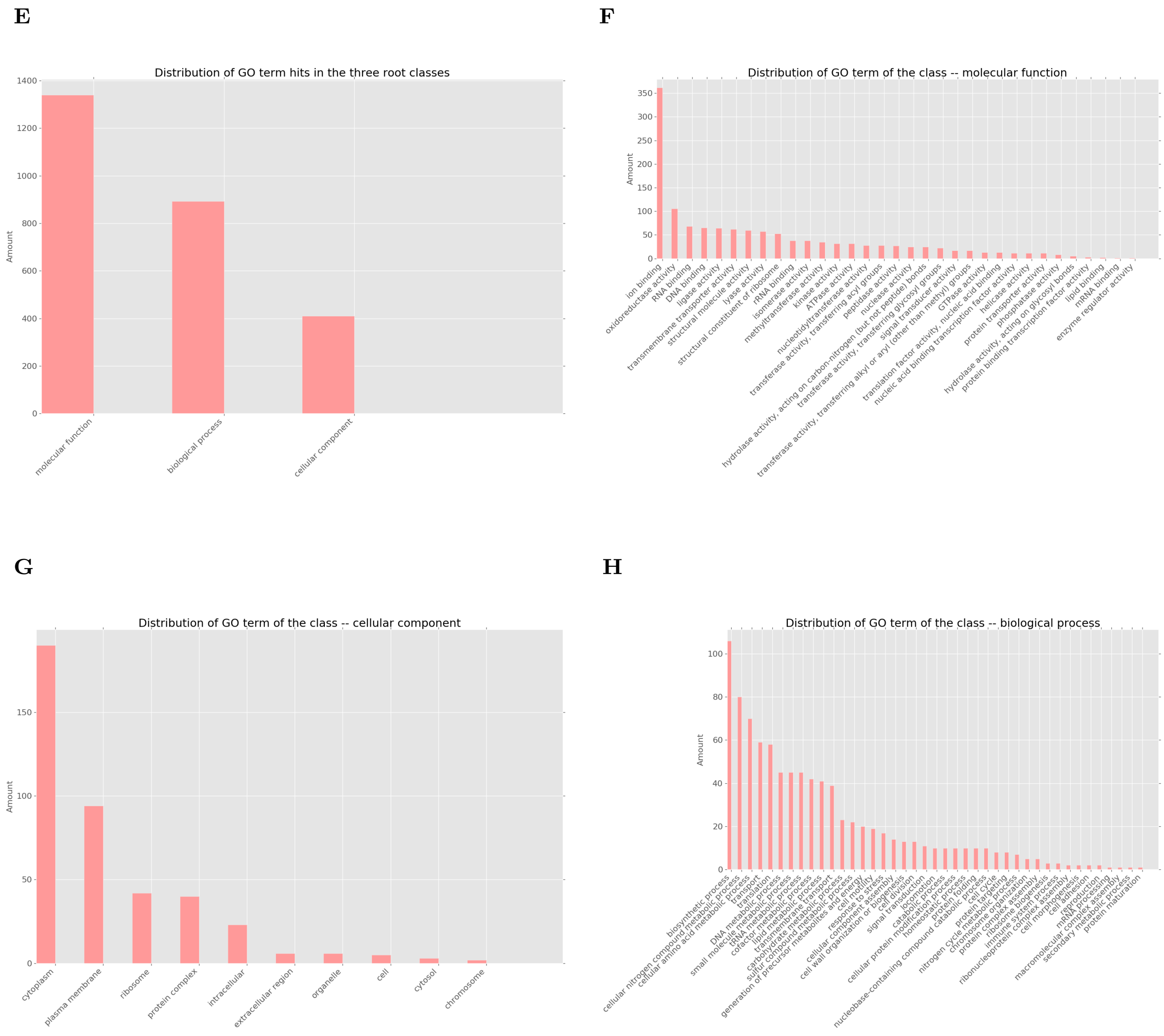

Figure S11: The distributions of GO term. Panels **(A)** to **(D)** display the distributions of three main domains, molecular function, cellular component and biological process of *Helicobacter pylori* 26695. Panels **(E)** to **(H)** show the same for *Campylobacter jejuni* 81116.

A

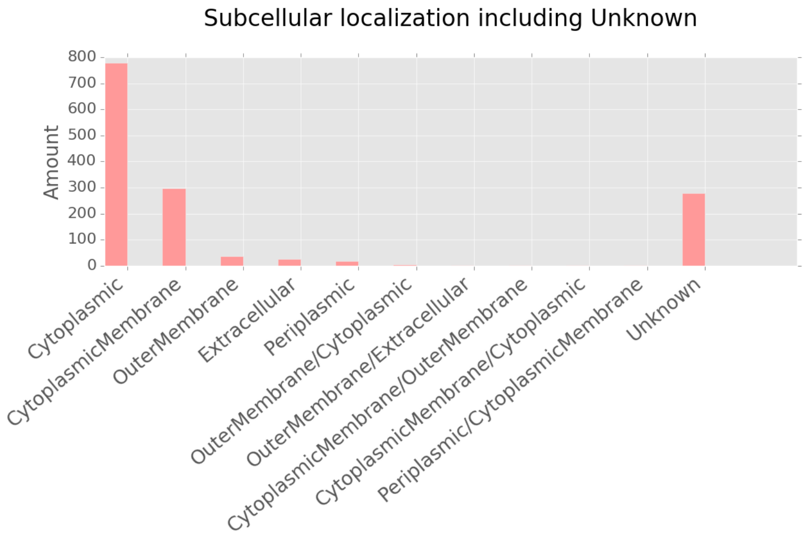

B

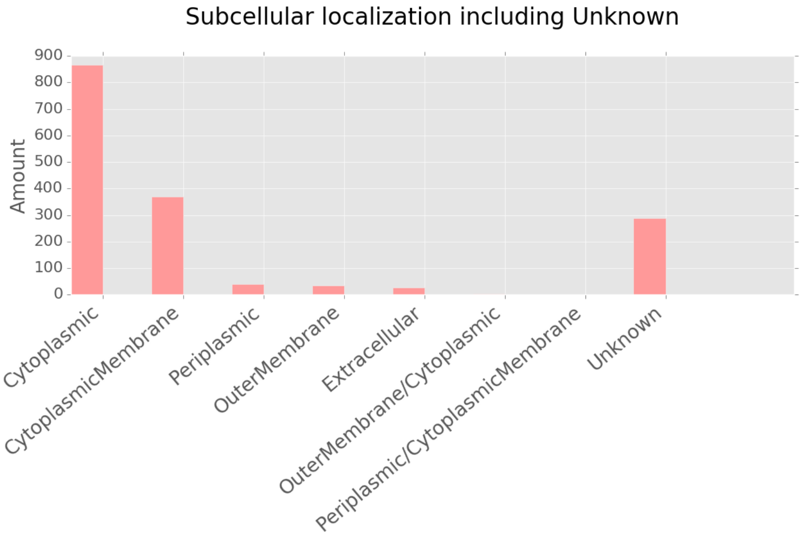

Figure S12: The distributions of subcellular localization of proteins for **(A)** *Helicobacter pylori* 26695, and **(B)** *Campylobacter jejuni* 81116.

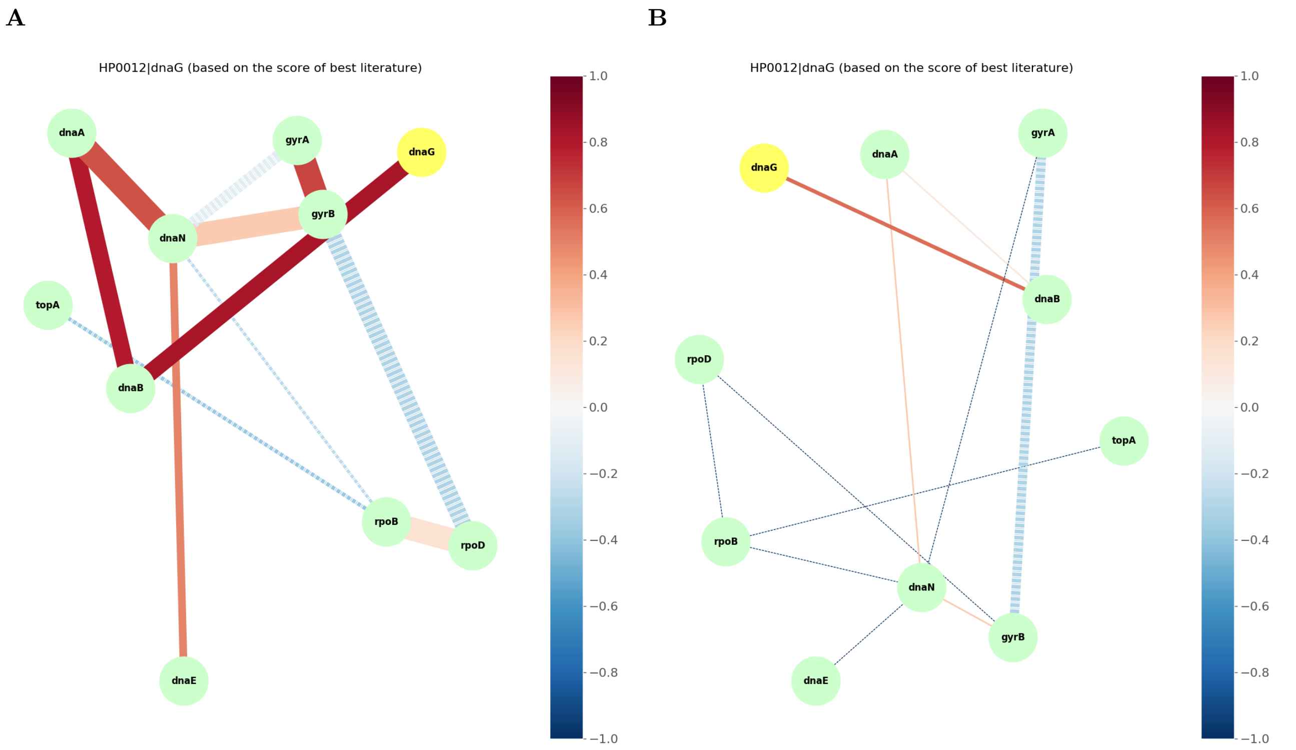

Figure S13: Visualization of protein-protein interactions. The yellow circles represent the query protein (dnaG) in *Helicobacter pylori* 26695. The other proteins are related to the query one showed as green circles. The dotted lines represent the interactions without support in the literature; the dash-dot lines represent the interactions with literature support but scores (given by PIE) below 0; the solid lines indicate that the interactions are supported in the literatures with high PIE score (higher than 0); the thickness of the lines is proportional to the number of articles that report the interaction; the color of connections encode score reported by PIE. **(A)** The result of search with the text "Helicobacter pylori" **(B)** The result of search with only protein names (without the strain name).

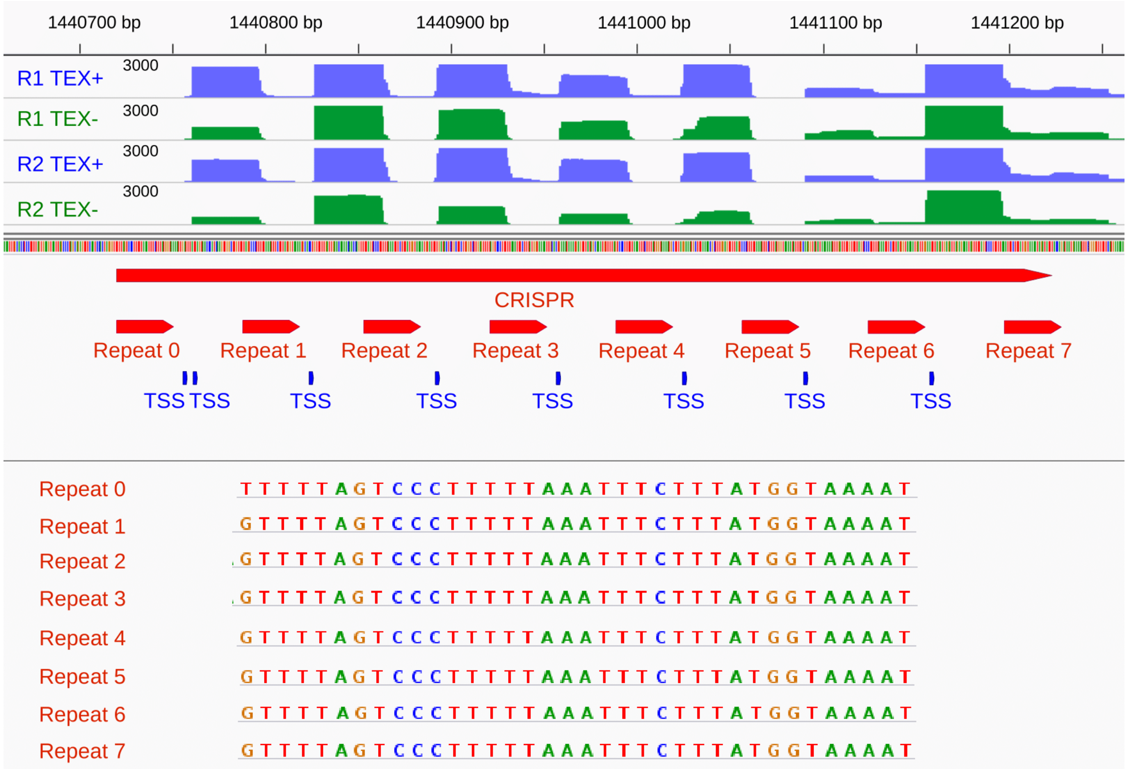

Figure S14: The example of CRISPR in *Campylobacter jejuni* 81116. The coverage of TEX+ and TEX- libraries of dRNA-Seq are presented as blue and green coverages, respectively. Red Bars represent CRISPR with repeat units, and Blue spots mean TSSs. Moreover, the repeat sequences are showed at the bottom.

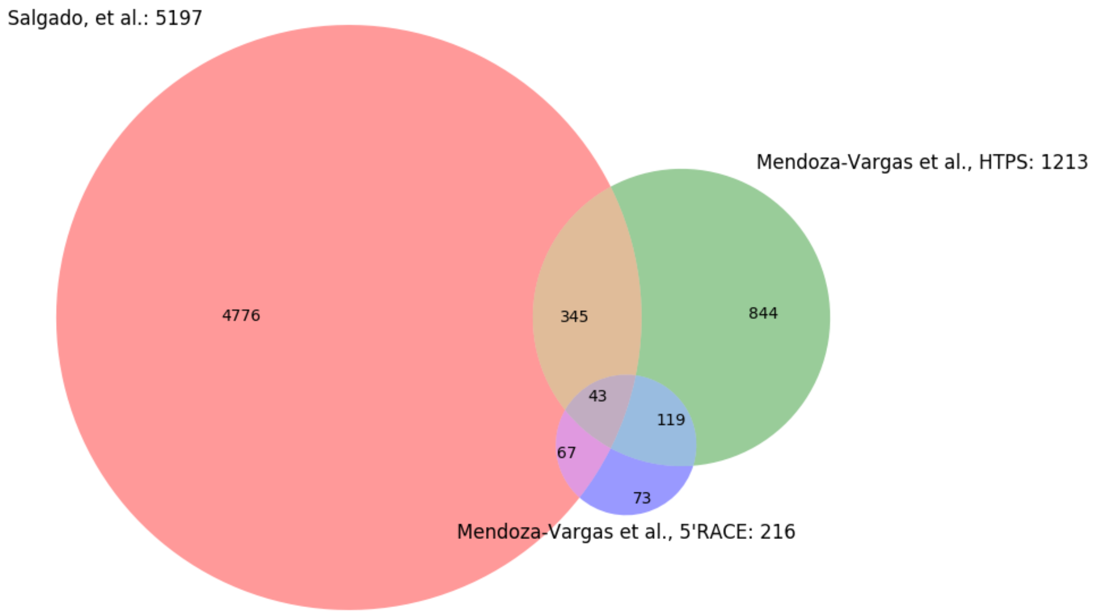

Figure S15: The overlap of three previously published TSS datasets in RegulonDB [1, 2].

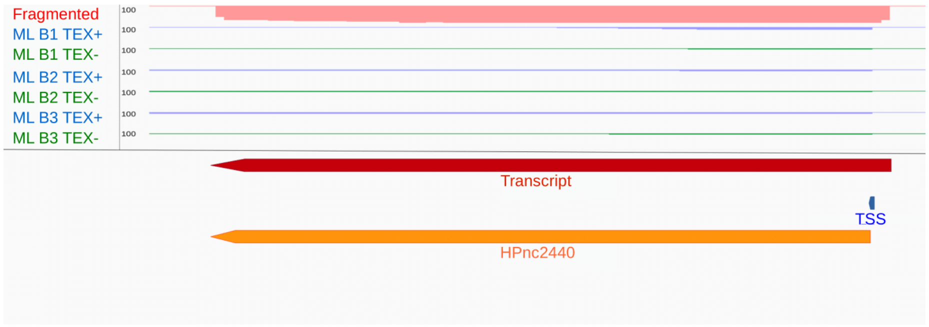

Figure S16: The predicted sRNA of *Helicobacter pylori* 26695 which can be detected only in data RNA-Seq after transcript fragmentation. The coverage of RNA-Seq with fragmentation, TEX+ and TEX- libraries of dRNA-Seq are presented as pink, blue and green coverages, respectively.

A

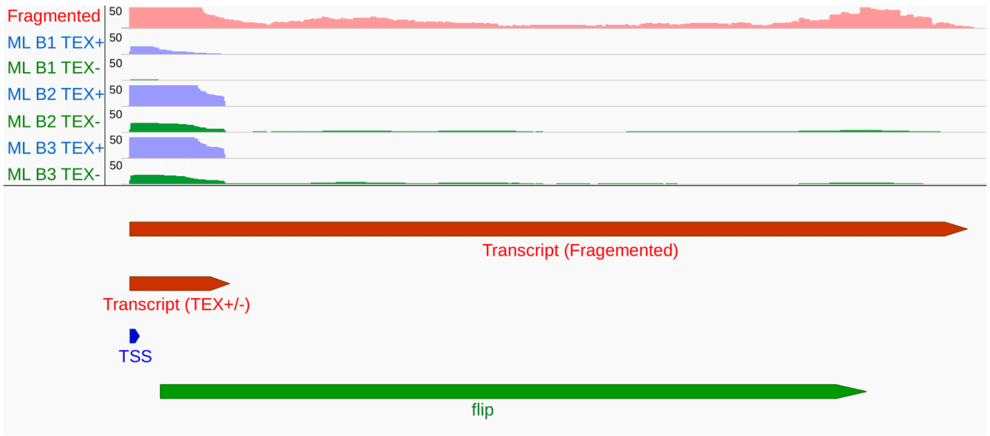

B

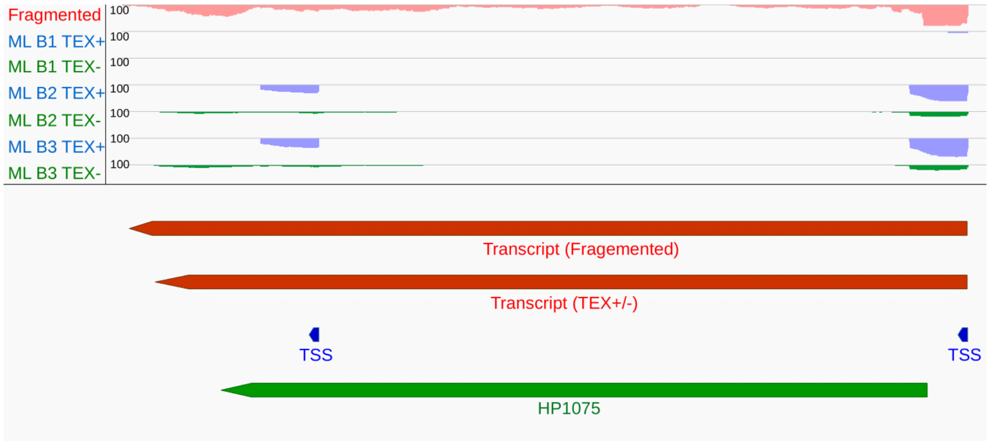

Figure S17: The comparison between dRNA-Seq and RNA-Seq after transcript fragmentation for detecting transcript in *Helicobacter pylori* 26695. The coverage of RNA-Seq with fragmentation, TEX+ and TEX- libraries of dRNA-Seq are presented as pink, blue and green coverages, respectively. Transcripts, TSSs, and genes (flip: 734056 - 734803 on the forward strand, HP1075: 1133935 - 1135251 on the reverse strand) are represented as red, blue and green bars, respectively. **(A)** Fragmented libraries are beneficial for detecting the 3' end of the transcript and the length of transcripts will be underestimated if only dRNA-Seq data is used. **(B)** The internal TSS predicted based on dRNA-Seq data is not even visually detectable in the RNA-Seq data.

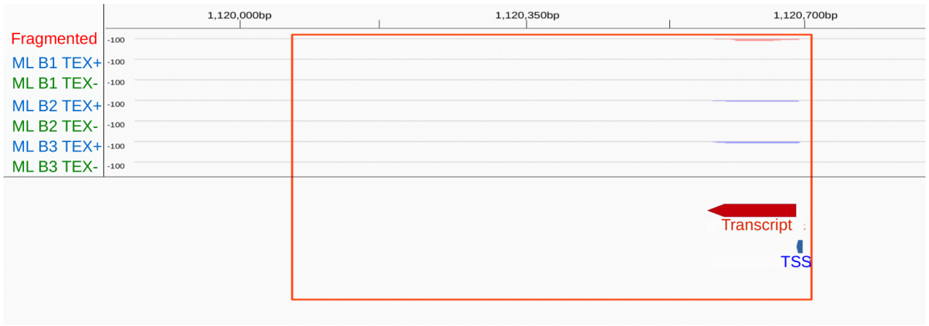

Figure S18: The lowly expressed sRNA of *Helicobacter pylori* 26695 (HPnc4610 – located in the region 968583 to 968616, orange hollow square) cannot be detected by ANNOgesic. The coverage of RNA-Seq with fragmentation, TEX+ and TEX- libraries of dRNA-Seq are presented as pink, blue and green coverages, respectively. TSS, CDS, and transcript are represented as blue, green and red bars, respectively. The average coverage of the low expressed benchmark is around 8 in the RNA-Seq data of the fragmented library and lower than 1 in the dRNA-Seq library.

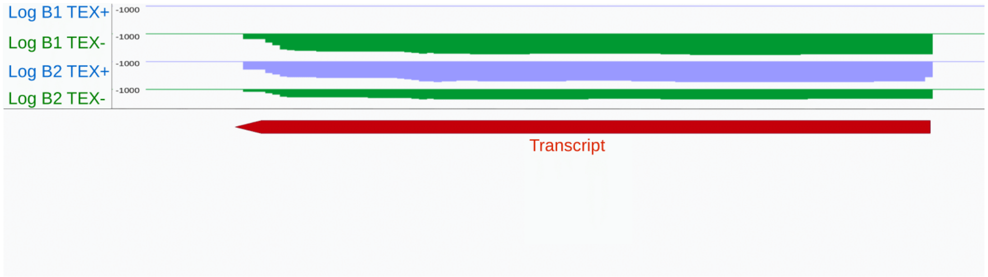

Figure S19: An example of known sRNA – CJnc230 of *Campylobacter jejuni* 81116 – which is not associated with a TSS as in one replicate the TEX+ library does not show sufficient coverage. The blue coverages shows the TEX+ libraries of dRNA-Seq and green coverages represents the TEX- libraries of dRNA-Seq.

Supplementary Tables

Table S1: The novelties and improvements of genomic feature detection in ANNOgesic

Feature	Approaches	Novelties
SNP	SAMtools [3] and BCFtools [3]	Filter of QUAL and read depth
TSS and processing site	TSSpredator [28]	Parameter optimization
CDS/tRNA/rRNA	RATT [5]	File format conversion
Transcript	New approach*	Detecting expressed region and modifying transcripts based on genome annotation
Terminator	TranstermHP [6] and a New approach	Coverage drop detection and checking structures of intergenic region between convergent genes
UTR	New approach	Comparison of TSSs, transcripts, CDSs, and terminators
Promoter	MEME [7] and GLAM2 [8]	Extraction of sequences automatically and TSS comparison
Operon	New approach	Comparison of TSSs, transcripts, CDSs, and terminators
sRNA	New approach	Detecting different types of sRNAs like UTR-derived sRNAs
sRNA target	RNAplex [9, 10], RNAup [9, 11] and IntaRNA [12]	Merging the results of RNAup, RNAplex and IntaRNA
sORF	New approach	Searching ORFs in transcripts with a RBS
Go term	Uniprot [13]	Comparison of transcripts
PPI network	STRING [14]	Network and Visualization with literature support by using PIE [15]
Subcellular localization	Psortb [16]	Comparison of transcripts
Circular RNA	Segamehl [17]	Comparison of genome annotation
Riboswitch and RNA thermometer	New approach	Extracting sequences with a RBS in UTRs for a infernal [19] search in Rfam [18]
CRISPR	CRT [20]	Comparison of genome annotation

*"New approach" means that the approach is newly developed in this study.

Table S2: The dependencies of the modules of ANNOgesic

Module	Dependencies	Version
Basic requirements	Python<br>BioPython<br>Wget<br>Matplotlib<br>Numpy	3.4<br>1.65<br>1.17.1<br>1.5.0<br>1.9.2
Annotation transfer	BioPerl<br>RATT	1.6.1<br>1.64
SNP calling	htslib<br>Samtools<br>Bcftools	1.3.1<br>1.3.1<br>1.3.1
TSS and PS	TSSpredator	1.06
TSS and PS parameter optimization	TSSpredator	1.06
sRNA	BLAST+<br>ViennaRNA (RNAfold)<br>nr database<br>BSRD database	2.2.28+<br>2.3.2<br>-<br>-
Terminator	TranstermHP<br>ViennaRNA (RNAfold)	2.09<br>2.3.2
Promoter	MEME<br>GLAM2<br>MPICH	4.11.1<br>4.11.1<br>3.2
sRNA target	ViennaRNA (RNAup, RNAplex, RNAplfold)<br>IntaRNA	2.3.2<br>2.0.4
Circular RNA	htslib<br>Samtools<br>Segemehl	1.3.1<br>1.3.1<br>0.1.9
Riboswitch and RNA thermometer	Infernal<br>Rfam	1.1.1<br>12.0
CRISPR	CRT	1.2
Subcellular localization	Psortb	3.0
Protein-protein interaction	Networkx<br>species.txt from STRING<br>PIE	1.10<br>9.1<br>-
GO term	idmapping_selected.tab from Uniprot<br>goslim.obo<br>go.obo	-<br>-<br>-
Screenshots of IGV	IGV	2.4
Colorization of screenshots	ImageMagick	6.9.0-0

All the used tools are either compatible with the ISC license of ANNOgesic or open source.

Table S3: The comparison between ANNOgesic predictions and several databases

Feature	Database	Sensitivity of *E. coli* from dRNA-Seq [29]	Sensitivity of *E. coli* from conventional RNA-Seq [30]	Sensitivity of *H. pylori* [27]	Sensitivity of *C. Jejuni* [28]
Transcript	EcoCyc [21]	86%	90%	-[i]	-
Operon	DOOR^2 [22]	72%	70%	74%	80%
	RegulonDB [23] [a]	90%	89%	-	-
sRNA[b]	RefSeq [24]	90%	70%	-[j]	-
	RegulonDB	80%	55%	-	-
	Others	-	-	90% [k]	84% [l]
TSS[c]	RegulonDB (3 datasets)	∼6%	-	-	-
Terminator[d]	RegulonDB	72%	70%	-	-
	EcoCyc	86%	84%	-	-
UTR[e]	RegulonDB	5' UTR 86%, 3' UTR 63%[f]	-	-	-
Promoter[g]	RegulonDB	39%	-	-	-
sORF[h]	Hemm *et. al* [25]	74%	-	-	-
Riboswitch	EcoCyc	83%	-	-	-
CRISPR	CRISPRdb [26]	100%	100%	100%	100%

[a]The features marked as "weak evidence" confidence level by RegulonDB were excluded.
[b]The non expressed sRNAs in published datasets were removed.
[c]The overlapped TSSs of three datasets are few. Moreover, most of the published TSSs (< 8%) are not associated with promoters.
[d]The terminators which do not contain coverage significant drop were removed.
[e]The non expressed UTRs in published datasets were excluded.
[f]The information of 3' end is usually lost in dRNA-Seq data.
[g]Based on TSSs information in the promoter set, only 22% promoters can be detected [7].
[h]The non expressed sORFs in published datasets were removed.
[i]"-" represents the feature of the strain has no proper dataset from the database or can not be generated.
[j]The sRNA comparison for *H. pylori* and *C. Jejuni* are done by other literature which shown in manuscript.
[k]sRNAs of *H. pylori* is from Sharma *et al.* [27].
[l]sRNAs of *C. Jejuni* is from Dugar *et al.* [28].

Table S4: The number of TSSs and their associated promoter motifs in RegulonDB [23]

Dataset	Total TSSs	Number of promoter motifs
Salgado *et al.* Illumina RNA-Seq [1]	5197	374 (7%)
Mendoza-Vargas *et al.* Roche 454 high-throughput pyrosequencing [2]	1213	23 (2%)
Mendoza-Vargas *et al.* Roche 5' RACE [2]	216	0 (0%)
TSSs of promoter set in RegulonDB	6478	1450 (22%)

References

[1] Salgado H., Peralta-Gil M., Gama-Castro S., Santos-Zavaleta A., Muñiz-Rascado L., García-Sotelo J. S., Weiss V., Solano-Lira H., Martínez-Flores I., Medina-Rivera A., Salgado-Osorio G., Alquicira-Hernández S., Alquicira-Hernández K., López-Fuentes A., Porrón-Sotelo L., Huerta A. M., Bonavides-Martínez C., Balderas-Martínez Y. I., Pannier L., Olvera M., Labastida A., Jiménez-Jacinto V., Vega-Alvarado L., del Moral-Chávez V., Hernández-Alvarez A., Morett E. and Collado-Vides J. (2013) RegulonDB v8.0: omics data sets, evolutionary conservation, regulatory phrases, cross-validated gold standards and more *Nucleic Acids Res.*, **41**, D203–D213.

[2] Mendoza-Vargas A., Olvera L., Olvera M., Grande R., Vega-Alvarado L., Taboada B., Jimenez-Jacinto V., Salgado H., Juárez K., Contreras-Moreira B., Huerta A. M., Collado-Vides J. and Morett E. (2009) Genome-Wide Identification of Transcription Start Sites, Promoters and Transcription Factor Binding Sites in *E. coli PLoS ONE*, **4**, e7526.

[3] Li H., Handsaker B., Wysoker A., Fennell T., Ruan J., Homer N., Marth G., Abecasis G., Durbin R. and 1000 Genome Project Data Processing Subgroup (2009) The *Sequence Alignment/Map* format and *SAMtools Bioinformatics*, **25**, 2078–2079.

[4] Dugar G., Herbig A., Förstner K.U., Heidrich N., Reinhardt R., Nieselt K. and Sharma C.M. (2013) High-resolution transcriptome maps reveal strain-specific regulatory features of multiple *Campylobacter* jejuni isolates *PLoS Genet.*, **9**, e1003495.

[5] Otto T.D., Dillon G.P., and Degrave W.S. and Berriman M. (2011) RATT: Rapid Annotation Transfer Tool *Nucleic Acids Res.*, **39**, e57.

[6] Kingsford C.L., Ayanbule K., and Salzberg S.L. (2007) Rapid, accurate, computational discovery of *Rho*-independent transcription terminators illuminates their relationship to *DNA* uptake *Genome Biol.*, **8**, R22.

[7] Bailey T.L., Williams N., Misleh C. and Li W.W. (2006) *MEME*: discovering and analyzing *DNA* and protein sequence motifs *Nucleic Acids Res.*, **34**, W369–373.

[8] Frith M.C., Saunders N.F.W., Kobe B. and Bailey T.L. (2008) Discovering Sequence Motifs with Arbitrary Insertions and Deletions *PLoS Comput. Biol.*, **4**, e1000071.

[9] Lorenz R., Bernhart S.H., Höner Zu Siederdissen C., Tafer H., Flamm C., Stadler P.F. and Hofacker I.L. (2011) *ViennaRNA Package* 2.0 *Algorithm. Mol. Biol.*, **6**, 26.

[10] Tafer H. and Hofacker I.L. (2008) *RNAplex*: a fast tool for *RNA-RNA* interaction search *Bioinformatics*, **24**, 2657–2663.

[11] Mückstein U., Tafer H., Hackermüller J., Bernhart S.H., Stadler P.F. and Hofacker I.L. (2006) Thermodynamics of *RNA-RNA* binding *Bioinformatics*, **22**, 1177–1182.

[12] Mann M., Wright P. R. and Backofen R. (2017) IntaRNA 2.0: enhanced and customizable prediction of RNA-RNA interactions *Nucleic Acids Res.*, **45**, W435–W439.

[13] Magrane M. and Uniprot Consortium (2011) *UniProt Knowledgebase*: a hub of integrated protein data *Database*, **2011**, bar009

[14] Szklarczyk D., Franceschini A., Wyder S., Forslund K., Heller D., Huerta-Cepas J., Simonovic M., Roth A., Santos A., Tsafou K. P., Kuhn M., Bork P., Jensen L. J., von Mering C. (2015) *STRING* v10: protein-protein interaction networks, integrated over the tree of life *Nucleic Acids Res.*, **43**, D447–452.

[15] Kim S., Shin S.Y., Lee I.H., Kim S.J., Sriram R. and Zhang B.T. (2008) *PIE*: an online prediction system for protein-protein interactions from text *Nucleic Acids Res.*, **36**, W411–415.

[16] Yu N.Y., Wagner J.R., Laird M.R., Melli G., Rey S., Lo R., Dao P., Sahinalp S.C., Ester M., Foster L.J. and Brinkman F.S.L. (2010) *PSORTb* 3.0: improved protein subcellular localization prediction with refined localization subcategories and predictive capabilities for all prokaryotes *Bioinformatics*, **26**, 1608–1615.

[17] Hoffmann S., Otto C., Doose G., Tanzer A., Langenberger D., Christ S., Kunz M., Holdt L.M., Teupser D., Hackermüller J. and Stadler P.F. (2014) A multi-split mapping algorithm for circular *RNA*, splicing, trans-splicing and fusion detection *Genome Biol.*, **15**, R34.

[18] Nawrocki E.P., Burge S.W., Bateman A., Daub J., Eberhardt R.Y., Eddy S.R., Floden E.W., Gardner P.P., Jones T.A., Tate J. and Finn R.D. (2014) Rfam 12.0: updates to the *RNA* families database *Nucleic Acids Res.*, **43**, D130–137.

[19] Nawrocki E.P. and Eddy S.R. (2013) Infernal 1.1: 100-fold faster *RNA* homology searches *Bioinformatics*, **29**, 2933–2935.

[20] Bland C., Ramsey T.L., Sabree F., Lowe M., Brown K., Kyrpides N.C. and Hugenholtz P. (2007) CRISPR recognition tool (CRT): a tool for automatic detection of clustered regularly interspaced palindromic repeats *BMC Bioinformatics*, **8**, 209.

[21] Keseler I. M., Collado-Vides J., Santos-Zavaleta A., Peralta-Gil M., Gama-Castro S., Muniz-Rascado L., Bonavides-Martinez C., Paley S., Krummenacker M., Altman T., Kaipa P., Spaulding A., Pacheco J., Latendresse M., Fulcher C., Sarker M., Shearer A. G., Mackie A., Paulsen I., Gunsalus R. P. and Karp P. D. (2011) EcoCyc: a comprehensive database of Escherichia coli biology *Nucleic Acids Res.*, **39**, D583–D590.

[22] Mao X., Ma Q., Zhou C., Chen X., Zhang H., Yang J., Mao F., Lai W. and Xu Y. (2014) DOOR 2.0: presenting operons and their functions through dynamic and integrated views *Nucleic Acids Res.*, **42**, D654–659.

[23] Gama-Castro S., Salgado H., Santos-Zavaleta A., Ledezma-Tejeida D., Muñiz-Rascado L., García-Sotelo J. S., Alquicira-Hernández K., Martínez-Flores I., Pannier L., Castro-Mondragón J. A., Medina-Rivera A., Solano-Lira H., Bonavides-Martínez C., Prez-Rueda E., Alquicira-Hernández S., Porrón-Sotelo L., López-Fuentes A., Hernández-Koutoucheva A., Del Moral-Chávez V., Rinaldi F. and Collado-Vides J. (2016) RegulonDB version 9.0: high-level integration of gene regulation, coexpression, motif clustering and beyond *Nucleic Acids Res.*, **44**, D133–143.

[24] Pruitt K. D., Tatusova T., Klimke W. and Maglott D. R. (2009) NCBI Reference Sequences: current status, policy and new initiatives *Nucleic Acids Res.*, **37**, D32–36.

[25] Hemm M. R., Paul B. J., Schneider T. D., Storz G. and Rudd K. E. (2008) Small membrane proteins found by comparative genomics and ribosome binding site models *Mol. Microbiol.*, **70**, 1487–1501.

[26] Grissa I., Vergnaud G. and Pourcel C. (2007) The CRISPRdb database and tools to display CRISPRs and to generate dictionaries of spacers and repeats *BMC Bioinformatics*, **8**, 172.

[27] Sharma C.M., Hoffmann S., Darfeuille F., Reignier, J., Findeiss S., Sittka A., Chabas S., Reiche K., Hackermüller J., Reinhardt R., Stadler P.F. and Vogel, J. (2010) The primary transcriptome of the major human pathogen *Helicobacter pylori Nature*, **464**, 250–255.

[28] Dugar G., Herbig A., Förstner K.U., Heidrich N., Reinhardt R., Nieselt K. and Sharma C.M. (2013) High-resolution transcriptome maps reveal strain-specific regulatory features of multiple *Campylobacter* jejuni isolates *PLoS Genet.*, **9**, e1003495.

[29] Thomason M. K., Bischler T., Eisenbart S. K., Förstner K. U., Zhang A., Herbig A., Nieselt K., Sharma C. M. and Storz G. (2015) Global Transcriptional Start Site Mapping Using Differential RNA Sequencing Reveals Novel Antisense RNAs in *Escherichia coli J. Bacteriol*, **197**, 18–28.

[30] McClure R., Balasubramanian D., Sun Y., Bobrovskyy M., Sumby P., Genco C.A., Vanderpool C.K. and Tjaden B. (2013) Computational analysis of bacterial RNA-seq data *Nucleic Acids Res.*, **41**, e140.
