# Supplementary material for: ANNOgesic: a Swiss army knife for the RNA-seq based annotation of bacterial/archaeal genomes
Source: Gigascience. 2018 Aug 30;7(9):giy096. doi: 10.1093/gigascience/giy096 (PMC6123526; doi:10.1093/gigascience/giy096)
Supplement: Response_to_Reviewer_Comments_Original_Submission.pdf [file giy096_response_to_reviewer_comments_original_submission.pdf]

Author's Response To Reviewer Comments

Close

See Rebuttal.pdf

Close
