# Supplementary material for: ANNOgesic: a Swiss army knife for the RNA-seq based annotation of bacterial/archaeal genomes
Source: Gigascience. 2018 Aug 30;7(9):giy096. doi: 10.1093/gigascience/giy096 (PMC6123526; doi:10.1093/gigascience/giy096)
Supplement: Response_to_Reviewer_Comments_Revision1.pdf [file giy096_response_to_reviewer_comments_revision1.pdf]

Author's Response To Reviewer Comments

Close

As wished we have inserted the citations of the GigaDB snapshot and the code ocean repository to the manuscript.

Close
