# Supplementary material for: ANNOgesic: a Swiss army knife for the RNA-seq based annotation of bacterial/archaeal genomes
Source: Gigascience. 2018 Aug 30;7(9):giy096. doi: 10.1093/gigascience/giy096 (PMC6123526; doi:10.1093/gigascience/giy096)
Supplement: Reviewer_1_Report_(Original_Submission) -- A. Rebecca Wattam — 02-26-2018 Reviewed [file giy096_reviewer_1_report_(original_submission).pdf]

Reviewer Report

Title: **ANNOgesic: A Swiss army knife for the RNA-Seq based annotation of bacterial/archaeal genomes**

Version: **Original Submission** **Date:** 2/26/2018

Reviewer name: **A. Rebecca Wattam**

Reviewer Comments to Author:

Sequencing data is a boon to science, but while biological researchers are taking advantage of it to answer more and more complex questions, they are often unable to construct the complicated pipelines necessary to analyze it. Yu et al. have constructed an analysis pipeline for RNA-Seq data. They string together a number of already developed tools to give the somewhat computationally trained researcher a number of options for analyzing their bacterial or archaeal reads. What these authors have done will benefit bacterial researchers. Their pipeline has default parameters, but also provides the ability to adjust the individual tools. Overall, both the pipeline and the manuscript are very nice.The manuscript is mostly well written but could use some adjustment of the English language. Specific examples are below:Page 2, lines 1-4. Should be changed to "Third party tools embedded into ANNOgesic are accessible via a consistent command-line interface."Page 2, line 24. Should be changed to "Depending on the task, ANNOgesic requires…"Page 3, line 11, second column. Should be changed to "Such analysis could be useful to generate…"Page 3, line 14, second column. Should be changed to "ANNOgesic can perform SNP/mutation calling via…"Page 5, line 23, second column. A comma is needed between 59 and 53.Page 5, line 44, second column. In the legend for Figure 5, shouldn't is say the coverage should not exceed a given cut-off coverage?The authors state that ANNOgesic requires information in wiggle format or BAM alignments, and suggests programs that can be used to generate this data. Why don't they just include this as part of the pipeline? This would make the pipeline more seamless for the computationally challenged.The authors state that certain modules require GFF3 format, but if not available, ANNOGesic will perform an annotation transfer from a closely related strain. It is unclear from the way it is written if the user is required to find this annotation and provide a different GFF3 file, or if ANNOgesic provides this.What are the limits to the number of data sets that can be compared? For example, there are 116 RNA-Seq data sets available in GEO for Escherichia coli MG1655. Could all of those be compared in ANNOgesic? Combining that number of datasets would provide deep insight into the dRNA of this strain.I can see in the supplementary figures that ANNOgesic summarizes the number of reads per each of the features that they predict. It would be good to mention this in the manuscript.On page 4, line 45, the authors state that they manually annotated TSSs in the first 200 kb of the H. pylori genome. Then, on line 48 they say the same thing. This needs to be edited.On page 4, line 3, second column the authors state that several transcripts undergo processing, which influences their biological activity. This sentence, and the associated paragraph, needs to be improved. Please define exactly what is meant by processing site, and how they can be manually annotated. Manually annotating TSS seems straight forward, but doing this for PS seems less so.Many of the references are missing page numbers, and end with ";."

Level of Interest

Please indicate how interesting you found the manuscript: An article of importance in its field

Quality of Written English

Please indicate the quality of language in the manuscript: Needs some language corrections before being published

Declaration of Competing Interests

Please complete a declaration of competing interests, considering the following questions:

- Have you in the past five years received reimbursements, fees, funding, or salary from an organisation that may in any way gain or lose financially from the publication of this manuscript, either now or in the future?
- Do you hold any stocks or shares in an organisation that may in any way gain or lose financially from the publication of this manuscript, either now or in the future?
- Do you hold or are you currently applying for any patents relating to the content of the manuscript?
- Have you received reimbursements, fees, funding, or salary from an organization that holds or has applied for patents relating to the content of the manuscript?
- Do you have any other financial competing interests?
- Do you have any non-financial competing interests in relation to this paper?

If you can answer no to all of the above, write 'I declare that I have no competing interests' below. If your reply is yes to any, please give details below.

I declare that I have no competing interests

I agree to the open peer review policy of the journal. I understand that my name will be included on my report to the authors and, if the manuscript is accepted for publication, my named report including any attachments I upload will be posted on the website along with the authors' responses. I agree for my report to be made available under an Open Access Creative Commons CC-BY license (http://creativecommons.org/licenses/by/4.0/). I understand that any comments which I do not wish to be included in my named report can be included as confidential comments to the editors, which will not be published.

I agree to the open peer review policy of the journal

To further support our reviewers, we have joined with Publons, where you can gain additional credit to further highlight your hard work (see: https://publons.com/journal/530/gigascience). On publication of this paper, your review will be automatically added to Publons, you can then choose whether or not to claim your Publons credit. I understand this statement.Yes
