# Supplementary material for: ANNOgesic: a Swiss army knife for the RNA-seq based annotation of bacterial/archaeal genomes
Source: Gigascience. 2018 Aug 30;7(9):giy096. doi: 10.1093/gigascience/giy096 (PMC6123526; doi:10.1093/gigascience/giy096)
Supplement: Reviewer_2_Report_(Original_Submission).pdf -- Raquel Tobes, Ph.D — 04-14-2018 Reviewed [file giy096_reviewer_2_report_(original_submission).pdf]

Reviewer Report

Title: **ANNOgesic: A Swiss army knife for the RNA-Seq based annotation of bacterial/archaeal genomes**

Version: **Original Submission** **Date:** 4/14/2018

Reviewer name: **Raquel Tobes, Ph.D**

Reviewer Comments to Author:

In this manuscript it is described a command-line tool called ANNOgesic that, based on RNA-Seq data and dRNA-Seq data, and using open bioinformatics tools designed for specific annotation tasks, is able to predict genome-wide features and to compile them into a consistent bacterial genome annotation.The features that ANNOgesic can annotate (if the needed data are provided as input) are:− Sequence modifications− SNPs/Mutations− Transcripts− Transcription Star Sites (TSS)− Terminators− UTRs− Processing sites (PS)− Promoters− Operons− Small RNAs (sRNA)− sRNA targets− Small ORFs (sORF)− GO term abundances− Protein-protein interaction networks− Subcellular localizations− Riboswitches− RNA thermometers− Circular RNA− CRISPR sequencesThe tool also includes a module for transferring annotations from a similar genome, some tools for annotating genomic features for which the authors have not found available tools and visualizations.ANNOgesic is open source software available under the ISC license.Minor concerns and improvementsThe annotation of some interesting features is based on dRNA-Seq results. This technology probably is not known by many readers and it would be useful to add some description of the methodology of analysis adding also some explanations about the results of sRNA-Seq that appear in many of the figures included in the manuscript.Figure 2 is crucial to introduce to the reader in the main concepts and features that the tool is able to analyze. I think that the size of this figure should be biger because many of the important elements are not easily identified.Given that this tool is a combination of a set of specific tools related with bacterial genome annotation it would be important to include in the manuscript a table with the tools and versions of the tools and databases that ANNOgesic uses for the analysis.The list taken from ANNOgesic documentation at http://annogesic.readthedocs.io/en/latest/required.html is followingRequired tools or databasesToolsBasic requirement:* Python : version higher or equal to 3.4.* BioPython: version higher or equal to 1.65.* Wget: version higher or equal to 1.17.1.* Matplotlib : version higher or equal to 1.5.0.Annotation transfer:* BioPerl: version higher or equal to 1.6.1.* RATT : version higher or equal to 1.64. SNP calling:* Samtools : version higher or equal to 1.3.1 (using htslib 1.3.1).* Bcftools : version higher or equal to 1.3.1 (using htslib 1.3.1).TSS and PS prediction:* TSSpredator : version higher or equal to 1.06.TSS and PS parameter optimization:* TSSpredator : version higher or equal to 1.06.sRNA detection:* Blast+ : version higher or equal to 2.2.28+.* ViennaRNA : version higher or equal to 2.3.2. RNAfold, mountain.pl and relplot.pl are needed for sRNA prediction.Terminator detection:* TranstermHP : version higher or equal to 2.09.* ViennaRNA : version higher or equal to 2.3.2. RNAfold is needed for terminator prediction.Promoter search:* MEME : version higher or equal to 4.11.1.* GLAM2 : version higher or equal to 4.11.1.* MPICH : version higher or equal to 3.2. It is for parallel version of promoter detection.sRNA target prediction:* ViennaRNA : version higher or equal to 2.3.2.* RNAup, RNAplex, RNAplfold are required for executing many modules of ANNOgesic.* IntaRNA: version higher or equal to 2.0.4.Circular RNA detection:* Samtools : version higher or equal to 1.3.1 (using htslib 1.3.1).* Segemehl : version higher or equal to 0.1.9. Riboswitch and RNA thermometer identification:* Infernal : version higher or equal to 1.1.1.CRISPR detection:* CRT: version higher or equal to 1.2.Subcellular localization prediction:* Psortb : version higher or equal to 3.0.* Protein-protein interaction detection:* Networkx : version higher or equal to 1.10.Generating screenshots of IGV:* IGVColorization of screenshots:* ImageMagick : version higher or equal to 6.9.0-0.DatabasessRNA detection:* BSRD (A suggestion for sRNA prediction and can be found here.)* nr databaseRiboswitch and RNA thermometer prediction:* Rfam (Only including Rfam IDs of riboswitches and RNA thermometers - the datasets can

be found here.)GO term identification:* idmapping_selected.tab from Uniprot* goslim.obo* go.oboProtein-protein interaction detection:* species.v${VERSION}.txt from STRING (${VERSION} represents the version number.)It would be important to mention that all the used tools are compatible with the ISC license of ANNOgesic.It would be useful to include the general requirements for using all the modules of the ANNOgesic tool.The table should also include the input files that are needed for each module.If the authors have developed some tools for annotating genomic features for which they had not found available tools and visualizations, these functionalities should be highlighted in the text. In parallel, all the integrative code or all the code oriented to analyze consistency of the different annotations tools compiled in ANNOgesic should be described in more detail.Taking into account that the tool has not any web application for non-bioinformatician users it would be useful to provide with one example with the needed code for running each of the modules that ANNOgesic tool includes.I agree with the authors that, given that high-level annotation including all the elements that ANNOgesic analyzes uses not to be available for bacterial genomes, to test the advantages of the annotations obtained using ANNOgesic is not easy. Hence, the feasibility and correction of the annotations depends on each of the component tools on which ANNOGESIC is based.Typos (between **):– For the chosen strains, H. pylori 26695 and C. jejuni 81116 annotation in GFF3 format were **be** obtained from NCBI RefSeq.– Nucleotide coverage data is used for **define** the expressed regions, and ….. – ANNOgesic is a **powful** tool for annotating numerous available....

Level of Interest

Please indicate how interesting you found the manuscript: Choose an item.

Quality of Written English

Please indicate the quality of language in the manuscript: Choose an item.

Declaration of Competing Interests

Please complete a declaration of competing interests, considering the following questions:

- Have you in the past five years received reimbursements, fees, funding, or salary from an organisation that may in any way gain or lose financially from the publication of this manuscript, either now or in the future?
- Do you hold any stocks or shares in an organisation that may in any way gain or lose financially from the publication of this manuscript, either now or in the future?
- Do you hold or are you currently applying for any patents relating to the content of the manuscript?
- Have you received reimbursements, fees, funding, or salary from an organization that holds or has applied for patents relating to the content of the manuscript?
- Do you have any other financial competing interests?
- Do you have any non-financial competing interests in relation to this paper?

If you can answer no to all of the above, write 'I declare that I have no competing interests' below. If your reply is yes to any, please give details below.

I declare that I have no competing interests

I agree to the open peer review policy of the journal. I understand that my name will be included on my report to the authors and, if the manuscript is accepted for publication, my named report including any attachments I upload will be posted on the website along with the authors' responses. I agree for my report to be made available under an Open Access Creative Commons CC-BY license (http://creativecommons.org/licenses/by/4.0/). I understand that any comments which I do not wish to be included in my named report can be included as confidential comments to the editors, which will not be published.

I agree to the open peer review policy of the journal

To further support our reviewers, we have joined with Publons, where you can gain additional credit to further highlight your hard work (see: https://publons.com/journal/530/gigascience). On publication of this paper, your review will be automatically added to Publons, you can then choose whether or not to claim your Publons credit. I understand this statement. Yes
