# Supplementary material for: ANNOgesic: a Swiss army knife for the RNA-seq based annotation of bacterial/archaeal genomes
Source: Gigascience. 2018 Aug 30;7(9):giy096. doi: 10.1093/gigascience/giy096 (PMC6123526; doi:10.1093/gigascience/giy096)
Supplement: Reviewer_3_Report_(Original_Submission).pdf -- Roy Chaudhuri — 07-17-2018 Reviewed [file giy096_reviewer_3_report_(original_submission).pdf]

Reviewer Report

Title: **ANNOgesic: A Swiss army knife for the RNA-Seq based annotation of bacterial/archaeal genomes**

Version: **Original Submission** **Date:** 4/17/2018

Reviewer name: **Roy Chaudhuri**

Reviewer Comments to Author:

The authors describe ANNOgesic, a command-line toolkit which wraps existing and novel tools to provide a standard interface for annotating bacterial and archaeal genome sequences using RNA-seq data. This is undoubtedly a useful piece of software which includes functionality not to my knowledge available via existing tools, and is of considerable interest to the community. The manuscript describes the software adequately and is mostly well written, I suggest just a few minor corrections:P2L7 was intensively tested -> has been intensively testedP4L47 benchmark the prediction -> benchmark the predictionsP6 (several places) Escherichia coli K12 -> Escherichia coli K-12P6L48 Here we presented -> Here we presentP7L17 an extensive documentation has been generated in order to guide the novice -> extensive documentation has been produced to guide noviceP7L24 ANNOgesic not only integrated the available tools, but also improve their performance -> ANNOgesic not only integrates the available tools, but also improves their performanceP7L28 A comprehensive documentation -> Comprehensive documentationLegends for tables S4-S7 manual-curated -> manually-curatedThe software seems to work as described. However I have several comments concerning the documentation and the software interface:In the installation instructions on readthedocs, the pip3 commands are a little brief, and two alternatives are provided without explanation. I suspect the command "pip3 install --user --upgrade ANNOgesic" would be more appropriate for most users.The installation instructions also include the unfinished sentence: "Only via the requirements are also installed."It is considered good practice to check for the presence of appropriate versions of software dependencies, and to give a helpful error message should they not be found (this is one of the ten recommendations for usable bioinformatics command-line tools published in GigaScience: https://gigascience.biomedcentral.com/articles/10.1186/2047-217X-2-15). ANNOgesic does not do this, and several times I ran into cryptic error messages which resulted from older versions of dependencies which were not easy to resolve (I ultimately had to upgrade bcftools, matplotlib and Java).On a similar note, it would be helpful if the wrapper scripts reported in full any system commands being run. That way, if there is any error with an external tool, it would be much more straightforward to identify.It would also be helpful if each tool reported a list of the full paths of the generated output files.Perhaps a log file could be kept storing all executed commands and their output for a particular analysis folder. This would greatly help with reproducability.Following the tutorial requires many additional downloads - perhaps consider a "download all command" which would download all required datasets (with the probable exception of nr for practical reasons). Alternatively an archive including all additional databases could be made available for download alongside the software.In the tutorial there is an error in the genome sequence retrieval command, which results in an error due to the presence of a second --ftp_path flag, which should be replaced with "--ref_gff"On readthedocs, several of the example commands in the tutorial are incorrectly formatted without a "code" tag, meaning that double hyphens do not display correctly.I had a problem with READemption - the realigned BAM files were not created automatically, meaning I had to manually convert the SAM files and index them using samtools.I also received an unhelpful error message when running the update_genome_fasta section of the tutorial: "There is a wrong path of -. Please check it!". This derived from the directory mutation_table not existing, meaning that the mv command creates a file named mutation_table. This would be fixed by adding the command "mkdir ANNOgesic/input/mutation_table" before the mv command.The "mutation_table" format used by update_genome_fasta seems to be a custom format. It would be preferable to use the widely-used VCF format to describe differences from the related genome.If optimize_tss_ps fails, subsequent runs fail with the message "Error: stat_$STRAIN.csv may be empty or has something wrong, please check it or remove it!!!". It would be more user-friendly to ignore

empty versions of this file. Additionally the error message should not contain the literal variable name "$STRAIN", but rather the correct filename and path.merge_features requires all gff files to be specified on the command line. Currently, if files are specified which do not exist, the rather unhelpful error message "Error: Some files in --other_features_files do not exist!" is returned - it would be more user-friendly to list the non-existent files in an error or (preferably) a non-fatal warning. It would also be helpful to have the option to automatically merge all gff files found in the output directory.

Level of Interest

Please indicate how interesting you found the manuscript: Choose an item.

Quality of Written English

Please indicate the quality of language in the manuscript: Choose an item.

Declaration of Competing Interests

Please complete a declaration of competing interests, considering the following questions:

- Have you in the past five years received reimbursements, fees, funding, or salary from an organisation that may in any way gain or lose financially from the publication of this manuscript, either now or in the future?
- Do you hold any stocks or shares in an organisation that may in any way gain or lose financially from the publication of this manuscript, either now or in the future?
- Do you hold or are you currently applying for any patents relating to the content of the manuscript?
- Have you received reimbursements, fees, funding, or salary from an organization that holds or has applied for patents relating to the content of the manuscript?
- Do you have any other financial competing interests?
- Do you have any non-financial competing interests in relation to this paper?

If you can answer no to all of the above, write 'I declare that I have no competing interests' below. If your reply is yes to any, please give details below.

I declare that I have no competing interests

I agree to the open peer review policy of the journal. I understand that my name will be included on my report to the authors and, if the manuscript is accepted for publication, my named report including any attachments I upload will be posted on the website along with the authors' responses. I agree for my report to be made available under an Open Access Creative Commons CC-BY license (http://creativecommons.org/licenses/by/4.0/). I understand that any comments which I do not wish to be included in my named report can be included as confidential comments to the editors, which will not be published.

I agree to the open peer review policy of the journal

To further support our reviewers, we have joined with Publons, where you can gain additional credit to further highlight your hard work (see: https://publons.com/journal/530/gigascience). On publication of this paper, your review will be automatically added to Publons, you can then choose whether or not to claim your Publons credit. I understand this statement. Yes
